# TrkB receptor interacts with mGlu_2_ receptor and mediates antipsychotic-like effects of mGlu_2_ receptor activation in the mouse

**DOI:** 10.1126/sciadv.adg1679

**Published:** 2024-01-26

**Authors:** Clémentine Eva Philibert, Candice Disdier, Pierre-André Lafon, Alexandre Bouyssou, Mathieu Oosterlaken, Sonya Galant, Anne Pizzoccaro, Pola Tuduri, Jeanne Ster, Jianfeng Liu, Julie Kniazeff, Jean-Philippe Pin, Philippe Rondard, Philippe Marin, Franck Vandermoere

**Affiliations:** ^1^ Institut de Génomique Fonctionnelle (IGF), Université de Montpellier, CNRS, INSERM, Montpellier, France.; ^2^ Cellular Signaling Laboratory, Key Laboratory of Molecular Biophysics of MOE, International Research Centre for Sensory Biology and Technology of MOST, College of Life Science and Technology, Huazhong University of Science and Technology, 430074 Wuhan, China.

## Abstract

Metabotropic glutamate receptor 2 (mGlu_2_) attracts particular attention as a possible target for a new class of antipsychotics. However, the signaling pathways transducing the effects of mGlu_2_ in the brain remain poorly characterized. Here, we addressed this issue by identifying native mGlu_2_ interactome in mouse prefrontal cortex. Nanobody-based affinity purification and mass spectrometry identified 149 candidate mGlu_2_ partners, including the neurotrophin receptor TrkB. The later interaction was confirmed both in cultured cells and prefrontal cortex. mGlu_2_ activation triggers phosphorylation of TrkB on Tyr^816^ in primary cortical neurons and prefrontal cortex. Reciprocally, TrkB stimulation enhances mGlu_2_-operated G_i/o_ protein activation. Furthermore, TrkB inhibition prevents the rescue of behavioral deficits by glutamatergic antipsychotics in phencyclidine-treated mice. Collectively, these results reveal a cross-talk between TrkB and mGlu_2_, which is key to the behavioral response to glutamatergic antipsychotics.

## Teaser

Deciphering mGlu_2_ interactome in vivo reveals interaction with TrkB, which is key to the response to glutamatergic antipsychotics.

## INTRODUCTION

Schizophrenia is a multifactorial and strongly debilitating mental disorder that affects around 1% of the world population. It is characterized by a broad range of symptoms classified as positive symptoms (e.g., hallucinations and disorder of thought), negative symptoms (e.g., social isolation and anhedonia), and cognitive deficits (e.g., poor “executive functioning” and impaired working memory) (*1*). Currently available antipsychotics that mainly target dopamine 2 (D_2_) and 5-HydroxyTryptamine/serotonin 2A (5-HT_2A_) receptors show unequivocal efficiency against positive symptoms. However, they poorly control negative and cognitive symptoms and induce severe side effects, such as long-term dyskinesia and metabolic perturbations (*2*). During the 90s, a different class of antipsychotics acting as agonists or positive allosteric modulators of group II metabotropic glutamate receptors (mGlu_2_ and mGlu_3_) has been designed (*3*, *4*). Subsequent preclinical studies indicated that mGlu_2_ activation mostly accounts for their antipsychotic activity (*5*, *6*), although implication of mGlu_3_ has also recently been reported (*7*). These glutamatergic antipsychotics displayed promising effects against positive, negative, and cognitive symptoms in preclinical studies (*8*), but leading compounds failed in phase 2 and phase 3 trials. Subsequent meta-analysis of clinical data revealed an interference with broadly prescribed atypical antipsychotics (*9*) that was further confirmed in preclinical studies, indicating that chronic pretreatment with atypical antipsychotics greatly reduces the antipsychotic activity of mGlu_2_ agonists (*10*). Accordingly, mGlu_2_ is still considered as a target for first line treatment of psychoses such as schizophrenia, and new molecules are under clinical evaluation.

mGlu_2_ is canonically coupled to G_i/o_ proteins that inhibit adenylyl cyclase (*11*) and calcium channels (*12*). However, the multiple signaling pathways transducing the effects of native mGlu_2_ and underlying its antipsychotic properties remain poorly characterized. Previous findings indicated that G protein–coupled receptor (GPCR)–operated signal transduction depends on their association with large protein networks. Thus, we characterized the interactome of native mGlu_2_ from mouse prefrontal cortex (PFC). This region expressing high amount of mGlu_2_ is involved in core symptoms of schizophrenia (*13*). We used a proteomic strategy combining mGlu_2_ affinity purification with a DN1 version of anti-mGlu_2_ nanobody (*14*) and systematic identification of copurified proteins by high-resolution mass spectrometry (MS) [affinity purification–MS (AP-MS)]. Among the 149 identified candidates, we focused on brain-derived neurotrophic factor (BDNF)/NT-3 growth factor receptor TrkB [also designated as neurotrophic tyrosine kinase receptor type 2 (NTRK2)]. Previous findings indicated a decreased expression of TrkB and its natural agonist BDNF in patients with schizophrenia and their involvement in the pathophysiology of schizophrenia (*15*). We explored the reciprocal influence of mGlu_2_ and TrkB in their signal transduction properties and the role of TrkB in the behavioral response to glutamatergic antipsychotics in phencyclidine (PCP)–treated mice, a rodent preclinical model of schizophrenia.

## RESULTS

### Analysis of mGlu_2_ interactome in mouse PFC identified 149 receptor-interacting proteins

**Fig. 1.**
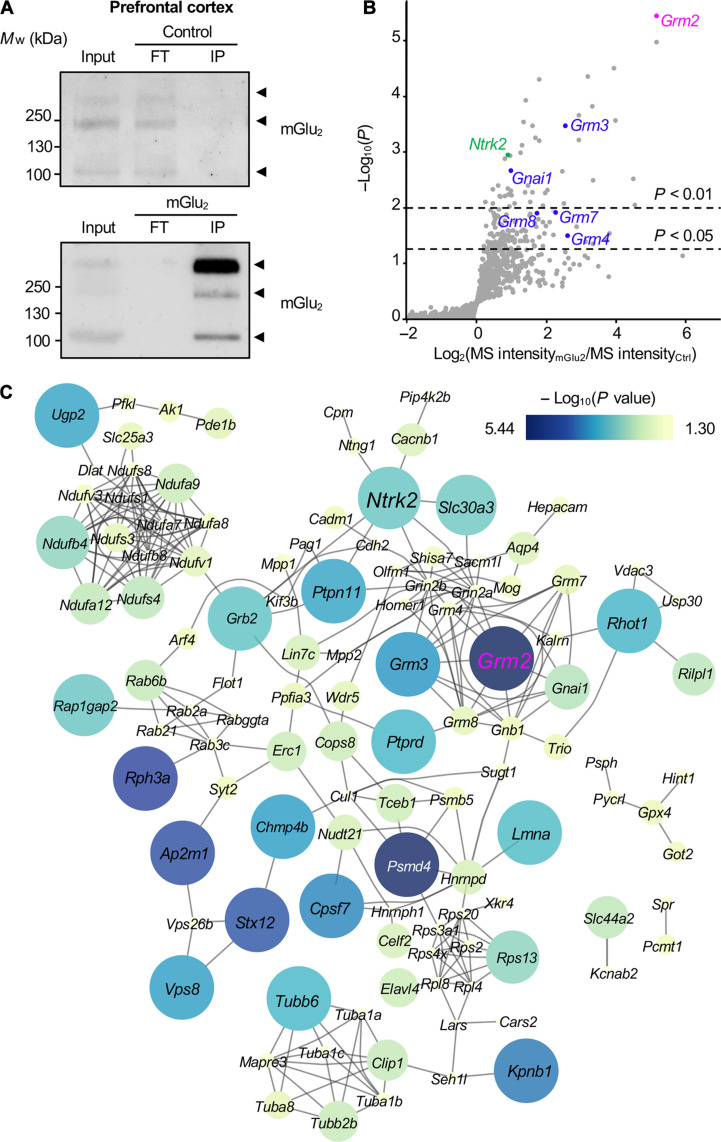
AP-MS analysis of mGlu_2_ interactome in mouse PFC. (**A**) Representative Western blots of immunoprecipitates obtained with a nontargeting nanobody (Ctrl) or an anti-mGlu_2_ nanobody (mGlu_2_) from mice PFC protein extracts. Inputs represent 5% of the material used for immunoprecipitations. mGlu_2_ was detected at three molecular weights that likely correspond to monomeric (100 kDa), dimeric (200 kDa), and higher–molecular weight complexes (more than 200 kDa), respectively. (**B**) Volcano plot illustrating the difference in protein abundance in mGlu_2_ immunoprecipitates versus Ctrl immunoprecipitates for each identified protein in three biological replicates. Proteins known to physically or functionally interact with mGlu_2_ (*GRM2*, pink) are highlighted in blue. TrkB (NTRK2) is highlighted in green. (**C**) STRING analysis of previously reported protein-protein interactions between proteins significantly enriched in mGlu_2_ immunoprecipitates (*P* < 0.05). Connecting lines represent previously reported protein-protein interactions in public databases. Size and color of nodes correspond to the statistical significance (−log_10_ of *P* value) of protein enrichment in mGlu_2_ immunoprecipitates. *M*_w_, weight-average molecular weight; IP, immunoprecipitation; FT, flow through of immunoprecipitation.

We used a nanobody specific for mGlu_2_ to immunoprecipitate endogenous receptor from mouse PFC, a brain structure involved in the effects of glutamatergic antipsychotics (*13*). This mGlu_2_ nanobody is a biparatopic nanobody composed of two previously described mGlu_2_-specific nanobodies that displays a subnanomolar affinity (0.30 ± 0.04 nM) for mGlu_2_ whatever its conformational state stabilized by a saturating concentration of either agonist or antagonist (*14*). The specificity of this nanobody for native mouse mGlu_2_ was assessed by immunohistochemistry experiments indicating a robust immunolabelling in the brain from wild-type mice, whereas no immunolabelling was detected in mGlu_2_ knockout (KO) (mGlu_2_^−/−^) mice (fig. S1A). This nanobody provided high immunoprecipitation yield, as shown by the absence of remaining receptors in brain extracts after immunoprecipitation with the mGlu_2_ nanobody (Fig. 1A).

MS analysis of proteins immunoprecipitated with a control nanobody that does not interact with mGlu_2_ and with the mGlu_2_ nanobody in three biological replicates (fig. S2A) identified 1736 proteins [false discovery rate (FDR) < 1%, available in PRIDE data repository]. Relative MS signal intensities of immunoprecipitated proteins showed that 149 proteins were significantly more abundant in the immunoprecipitates obtained with the mGlu_2_ nanobody than in the immunoprecipitates with the control nanobody (*P* < 0.05; Fig. 1B and table S1). As expected, mGlu_2_ (*GRM2*, highlighted in pink, Fig. 1B) was the most significantly enriched protein in immunoprecipitates obtained with the mGlu_2_ nanobody (*P* = 1 × 10^−5.44^). Other mGlu receptors known to be expressed in PFC and to form heteromers with mGlu_2_ (mGlu_3_, mGlu_4_, mGlu_7_, and mGlu_8_) (*16*) and subunits of the canonically mGlu_2_-coupled G_i/o_ proteins (Gα_i1_ and Gβγ subunits) (*17*) were also enriched in mGlu_2_ immunoprecipitates (Fig. 1B), thus validating our nanobody-based strategy to identify the mGlu_2_ interactome.

Analysis of previously described associations between mGlu_2_ candidate partners using String showed that 138 of 149 proteins enriched in mGlu_2_ immunoprecipitate were physically connected with at least one other mGlu_2_ candidate partner. One hundred nine of them are part of a unique complex (Fig. 1C). Gene Ontology (GO) (*18*) analysis of this highly connected complex using the 1736 proteins identified by MS/MS as reference protein list showed a strong enrichment in biological process and molecular function GO terms relevant to mGlu_2_ functions such as “glutamate receptor signaling pathway” (*P* = 1.979 × 10^−08^), “adenylate cyclase-inhibiting G protein–coupled glutamate receptor signaling pathway” (*P* = 2.960 × 10^−08^), “regulation of synaptic transmission, glutamatergic” (*P* = 3.523 × 10^−05^), “glutamate receptor activity” (*P* = 1.015 × 10^−09^), and “adenylate cyclase inhibiting G protein–coupled glutamate receptor activity” (*P* = 3.397 × 10^−07^) (table S2 and fig. S2B). Analysis of cellular component annotations indicated a strong enrichment in “presynaptic active zone” (*P* = 1.743 × 10^−8^), “intrinsic component of presynaptic membrane” (*P* = 6.414 × 10^−6^), “presynaptic membrane” (*P* = 1.337 × 10^−5^), “integral component of presynaptic membrane” (*P* = 6.099 × 10^−5^), and “intrinsic component of presynaptic active zone membrane” (*P* = 8.240 × 10^−4^) GO terms (table S2), consistent with the predominant presynaptic localization of mGlu_2_ (*19*).

Among proteins showing the highest enrichment in the mGlu_2_ interactome, we focused on BDNF/NT-3 growth factor receptor TrkB (also called *NTRK2*, highlighted in green), given the well-documented influence of the BDNF/TrkB axis on the pathophysiology of schizophrenia (Fig. 1B and table S1) (*15*).

### TrkB specifically and dynamically associates with mGlu_2_

**Fig. 2.**
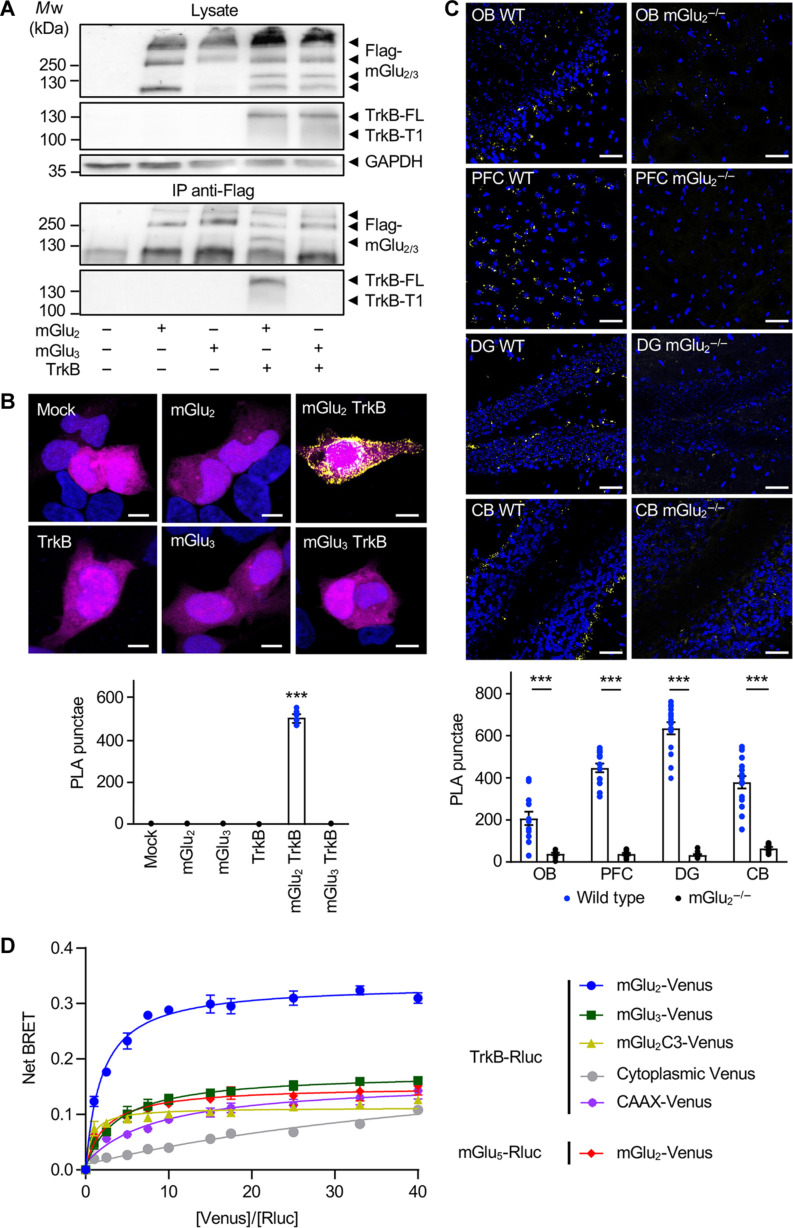
TrkB interacts with mGlu_2_ but not with mGlu_3_. (**A**) Western blots of anti-Flag immunoprecipitates obtained from HEK-293 cells expressing human Flag-tagged mGlu_2_ or mGlu_3_ in the absence or presence of human TrkB. Inputs (lysate) and anti-Flag immunoprecipitates (IP) were probed with anti-Flag antibody to detect mGlu_2_ and mGlu_3_, anti-TrkB antibody, and with anti-GAPDH antibody. The data are representative of four independent experiments performed on different cultures. (**B**) Representative images of PLA experiments performed with anti-Flag and anti-TrkB antibodies in HEK-293 cells expressing either human Flag-tagged mGlu_2_ or mGlu_3_ in the absence or presence of human TrkB. PLA signal is shown in yellow, cell nuclei are stained with 4′,6-diamidino-2-phenylindole (DAPI) in blue, and green fluorescent protein transfection control is shown in magenta. Scale bars, 5 μm. Data are representative of four independent experiments performed on different cultures. (**C**) Representative images of PLA experiments performed with the anti-mGlu_2_ nanobody and the anti-TrkB antibody on OB, PFC, DG, and CB slices from wild-type (WT) or mGlu_2_ KO (mGlu_2_^−/−^) mice. PLA signal is shown in yellow; cell nuclei are stained with DAPI in blue. Scale bars, 50 μm. The histogram shows means ± SEM of total PLA punctae per region of interest from 10 to 13 mice per condition. Two-way analysis of variance (ANOVA) (compared between wild type and mGlu_2_^−/−^, post hoc Šidák’s test): ***P < 0.001. (**D**) BRET saturation curves obtained from HEK-293 cells expressing a fixed amount of Rluc-TrkB or Rluc-mGlu_5_ and increasing amounts of Venus-mGlu_2_ or Venus-mGlu_3_ or Venus-mGlu_2_C3 (mGlu_2_ with mGlu_3_ C-terminal) or membrane bound CAAX-Venus or cytoplasmic Venus. Curves show means ± SEM of net BRET signals measured in three technical replicates of one experiment representative of four biological replicates. Two-way ANOVA (post hoc Šidák’s test): *F*_8,26_ = 638.6, ***P < 0.001.

Co-immunoprecipitation followed by Western blotting showed that TrkB associates with mGlu_2_ in PFC from wild-type mice, but not in mGlu_2_^−/−^ mice (fig. S1B), confirming the specificity of our nanobody-based interactomic screen. To further explore the specificity of mGlu_2_ interaction with TrkB, human TrkB was coexpressed with either human Flag-tagged mGlu_2_ or the closely related mGlu_3_ in human embryonic kidney (HEK) 293 cells. Immunoprecipitation of mGlu receptors with a Flag antibody followed by Western blotting confirmed interaction of TrkB with mGlu_2_, while no association of TrkB with mGlu_3_ was observed (Fig. 2A). Consistent with these results and further suggesting a specific association of TrkB with mGlu_2_, PLA with anti-TrkB and anti-Flag antibodies showed a PLA signal only in cells coexpressing TrkB and Flag-mGlu_2_ but not in cells coexpressing TrkB and Flag-mGlu_3_ (Fig. 2B). No signal was detected in cells transfected with empty vectors (Mock) or expressing either Flag-mGlu_2_ or Flag-mGlu_3_ or TrkB alone (Fig. 2B). Likewise, in situ PLA performed with the anti-mGlu_2_ nanobody and the anti-TrkB antibody showed a close proximity of the endogenous receptors in various brain regions from the mouse known to coexpress both receptors, such as the olfactory bulb (OB), PFC, dentate gyrus (DG), and cerebellum (CB) (Fig. 2C) (*20*, *21*). PLA signals were sparse and of lower intensity in the brain from mGlu_2_^−/−^ mice (Fig. 2C).

We next assessed interaction between TrkB and mGlu_2_ or mGlu_3_ using bioluminescence resonance energy transfer (BRET). Under conditions of constant TrkB-Rluc expression, the BRET signal increased hyperbolically as a function of mGlu_2_-Venus expression (Fig. 2D), indicative of an interaction between both receptors. In contrast and further supporting the notion that TrkB does not associate with mGlu_3_, the BRET saturation curve in cells coexpressing TrkB-Rluc and mGlu_3_-Venus was comparable to those observed in cells coexpressing mGlu_5_-Rluc and mGlu_2_-Venus, two receptors that do not heteromerize (*16*), and in cells coexpressing TrkB-Rluc + CAAX-Venus as control membrane protein (Fig. 2D). The BRET saturation curve in cells coexpressing TrkB-Rluc and mGlu_2_C3-Venus, an mGlu_2_ in which the C-terminal domain was replaced by C-terminal domain of mGlu_3_, was also comparable to the one observed in TrkB-Rluc + CAAX-Venus–expressing cells (Fig. 2D), indicating that the C-terminal domain of mGlu_2_ is necessary for its interaction with TrkB. Note that a linear shape of the BRET curve was only observed in control cells coexpressing TrkB-Rluc and cytoplasmic Venus.

**Fig. 3.**
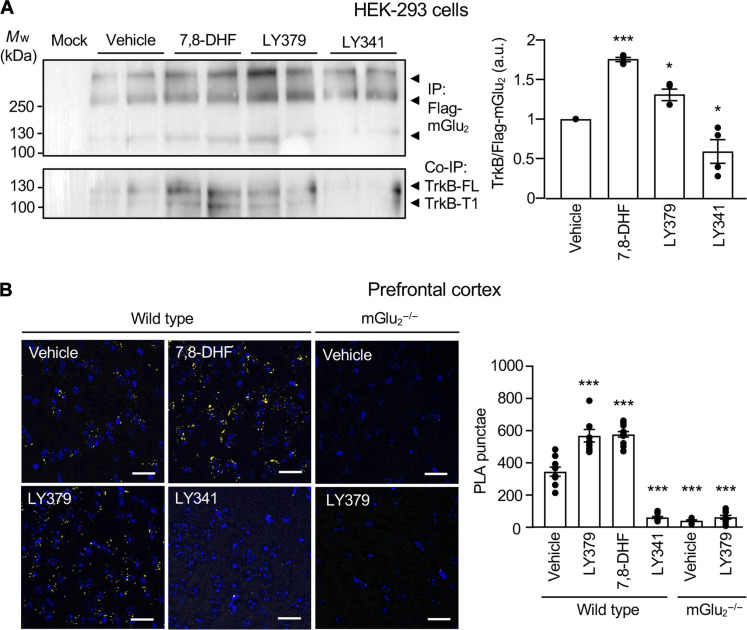
mGlu_2_ and TrkB conformational states dynamically modulate mGlu_2_/TrkB interaction. (**A**) Representative Western blot of anti-Flag immunoprecipitates performed from HEK-293 cells coexpressing human Flag-tagged mGlu_2_ and TrkB. Cells were treated for 15 min with either vehicle (Veh) or 7,8-DHF (1 μM) or LY379268 (1 μM; LY379) or LY341495 (3 μM; LY341). The histogram represents the means ± SEM of anti-TrkB immunoreactive signal normalized to anti-Flag immunoreactive signal [in arbitrary units (a.u.), *n* = 4]. One-way ANOVA (multiple comparisons compared to vehicle, post hoc Dunnett’s test): *F*_3,16_ = 1.264, *P < 0.05, and ***P < 0.001. (**B**) Representative images of PLA experiments performed with the anti-mGlu_2_ nanobody and the anti-TrkB antibody on PFC slices from wild-type or mGlu_2_^−/−^ mice. Wild-type mice were injected with either vehicle or LY379268 (10 mg/kg; LY379) or 7,8-DHF (5 mg/kg) or LY341495 (3 mg/kg; LY341). mGlu_2_^−/−^ mice were injected either with vehicle or LY379268. PLA signal is shown in yellow; cell nuclei are stained with DAPI (blue). Scale bars, 25 μm. Histograms represent the means ± SEM of the number of PLA punctae in PFC from 8 to 11 animals per condition. Two-way ANOVA (multiple comparisons compared to wild-type vehicle, post hoc Dunnett’s test): *F*_5,49_ = 130.9, *P < 0.05 and ***P < 0.001.

Collectively, these observations indicate that TrkB specifically associates with mGlu_2_, but not with the closely related mGlu_3_, and that this interaction already occurs in absence of agonist of either receptor. Treatment of HEK-293 cells coexpressing Flag-mGlu_2_ and TrkB with either the mGlu_2_ agonist LY379268 (1 μM) or the TrkB agonist 7,8-dihydroxyflavone (7,8-DHF) (1 μM) increased the interaction between both receptors, as assessed by co-immunoprecipitation (Fig. 3A). In contrast, exposing cells with the mGlu_2_ antagonist LY341495 (10 μM) reduced TrkB co-immunoprecipitation with Flag-mGlu_2_ (Fig. 3A). LY379268 and 7,8-DHF also increased the formation of mGlu_2_-TrkB complexes in a concentration-dependent manner in primary cortical neurons (fig. S3). Corroborating our observations in cell cultures, injection of wild-type mice with LY379268 (10 mg/kg, 30 min, i.p.) or 7,8-DHF (5 mg/kg, 1 hour, i.p.) increased the number of PLA punctae in PFC compared with vehicle-injected mice, whereas LY341495 (3 mg/kg, 30 min, i.p.) injection caused a strong decrease in PLA signals (Fig 3A). These results suggest that mGlu_2_-TrkB interaction is a dynamic process that depends on their activation state. As expected, LY379268 administration to mGlu_2_^−/−^ mice did not increase the low number of PLA punctae in PFC (Fig. 3B).

### mGlu_2_ stimulation transactivates TrkB

**Fig. 4.**
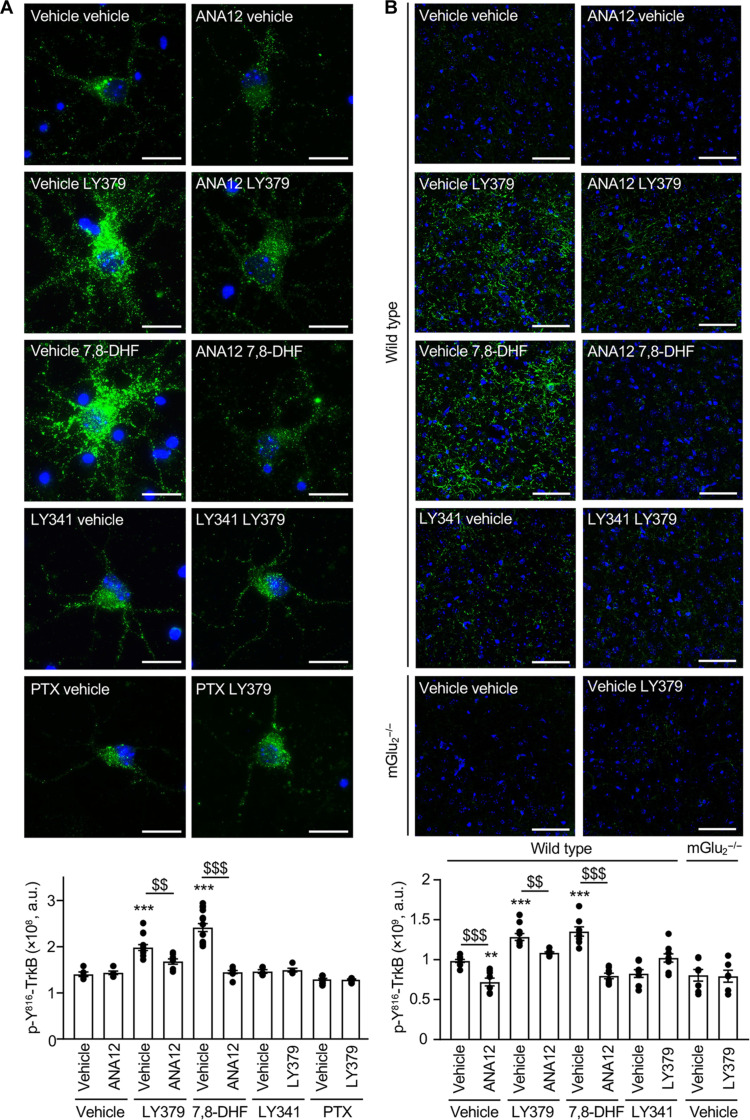
mGlu_2_ stimulation transactivates TrkB. (**A**) Representative images of p–Y^816^-TrkB immunolabeling (green) in four independent cultures of cortical neurons. Cells were pretreated with either vehicle or ANA-12 (10 μM) or LY341495 (10 μM; LY341) or PTX (0.2 μg/ml) and then challenged for 15 min with either vehicle or LY379268 (1 μM; LY379) or 7,8-DHF (1 μM). The histogram represents the means ± SEM of anti–p–Y^816^-TrkB immunoreactive signal (expressed as the number of positive pixels per field, *n* = 7). Scale bars, 20 μm. One-way ANOVA (compared to the associated vehicle pretreatment, post hoc Šidák’s test): *F*_9,79_ = 6.110, $$*P* < 0.005, and $$$*P* < 0.001. One-way ANOVA (multiple comparisons compared to vehicle vehicle, post hoc Dunnett’s test): *F*_9,79_ = 6.110, **P < 0.005, and ***P < 0.001. (**B**) Representative images of p–Y^816^-TrkB immunolabeling (green) in coronal PFC slices from 6 to 10 mice per condition. Wild-type mice were pretreated with either vehicle or ANA-12 (2.5 mg/kg) or LY341495 (3 mg/kg; LY341) and then injected with either vehicle or LY379268 (10 mg/kg; LY379) or 7,8-DHF (5 mg/kg). mGlu_2_^−/−^ mice were injected with either vehicle or LY379268. The histogram represents the means ± SEM of anti–p–Y^816^-TrkB immunoreactive signal (expressed as the number of positive pixels per field) measured after the indicated treatments. Scale bars, 50 μm. One-way ANOVA compared to the associated vehicle pretreatment (post hoc Šidák’s test): *F*_9,70_ = 2.309, $$*P* < 0.005, and $$$*P* < 0.001. One-way ANOVA (multiple comparisons compared to vehicle vehicle, post hoc Dunnett’s test): *F*_9,70_ = 2.309, **P < 0.005, and ***P < 0.001.

In light of growing evidence indicating that GPCRs can transactivate receptor tyrosine kinases (RTKs) (*22*), we examined the impact of mGlu_2_ stimulation on TrkB activation by measuring its phosphorylation status on Tyr^816^(p–Y^816^-TrkB). Exposing primary cortical neurons to LY379268 (1 μM) increased phospho p–Y^816^-TrkB, as shown by immunocytochemistry (Fig. 4A) and Western blotting (figs. S4 and S5). Increase in TrkB phosphorylation was detected 2 min after the onset of the treatment, reached a maximum level at 10 min, and decreased after 15 min (fig. S4). It was already observed in neurons exposed for 15 min to 10 nM LY379268 and reached a maximal level at 100 nM (fig. S5). LY379268 exposure of cortical neurons also induced a concentration-dependent increase in phospholipase Cγ1 phosphorylation on Tyr^783^ (p–Y^783^-PLCγ1), a direct substrate of TrkB (fig. S5). The LY379268 effect was abolished by pretreating cultures with either LY341495 (10 μM) or N-[2-[[(hexahydro-2-oxo-1H-azepin-3-yl)amino]carbonyl]phenyl]benzo[b]thiophene-2-carboxamide [(ANA12), 10 μM], a TrkB antagonist (Fig. 4A). As expected, exposing cultures to 7,8-DHF also enhanced p–Y^816^-TrkB, an effect abolished by pretreatment of neurons with ANA-12. Pretreating neurons with pertussis toxin (PTX; 0.2 μg/ml) prevented LY379268-induced increase in TrkB phosphorylation (Fig. 4A), indicating the involvement of Gα_i/o_ protein in TrkB transactivation by mGlu_2_. Furthermore, the LY379268-induced increase in TrkB phosphorylation was not prevented by exposing neurons to a BDNF neutralizing antibody, which did inhibit TrkB activation elicited by exogenously added BDNF (10 nM, fig. S6).

Administration of LY379268 (10 mg/kg, 30 min, i.p.) likewise increased p–Y^816^-TrkB immunolabeling in PFC of wild-type mice, but not mGlu_2_^−/−^ mice (Fig. 4B). This increase in TrkB phosphorylation reached a level comparable to that measured in mice injected with 7,8-DHF (5 mg/kg, 1 hour, i.p.) (Fig. 4B). Reminiscent to our observations in cultured neurons, the LY379268 effect was abolished by pretreating mice with LY341495 (3 mg/kg, 30 min, i.p.) or with ANA-12 (2.5 mg/kg, 2 hours, i.p.). Administration of ANA-12 also prevented the increase in p–Y^816^-TrkB immunolabeling elicited by 7,8-DHF injection. Collectively, these results indicate that mGlu_2_ stimulation transactivates TrkB in neurons from PFC. These findings were confirmed by Western blotting experiments showing that administration of LY379268 (0.01 to 10 mg/kg, 30 min, i.p.,) to mice induced a dose-dependent increase in p–Y^816^-TrkB and p–Y^783^-PLCγ1 (fig. S5).

### TrkB stimulation enhances mGlu_2_ coupling to G_i/o_ proteins

**Fig. 5.**
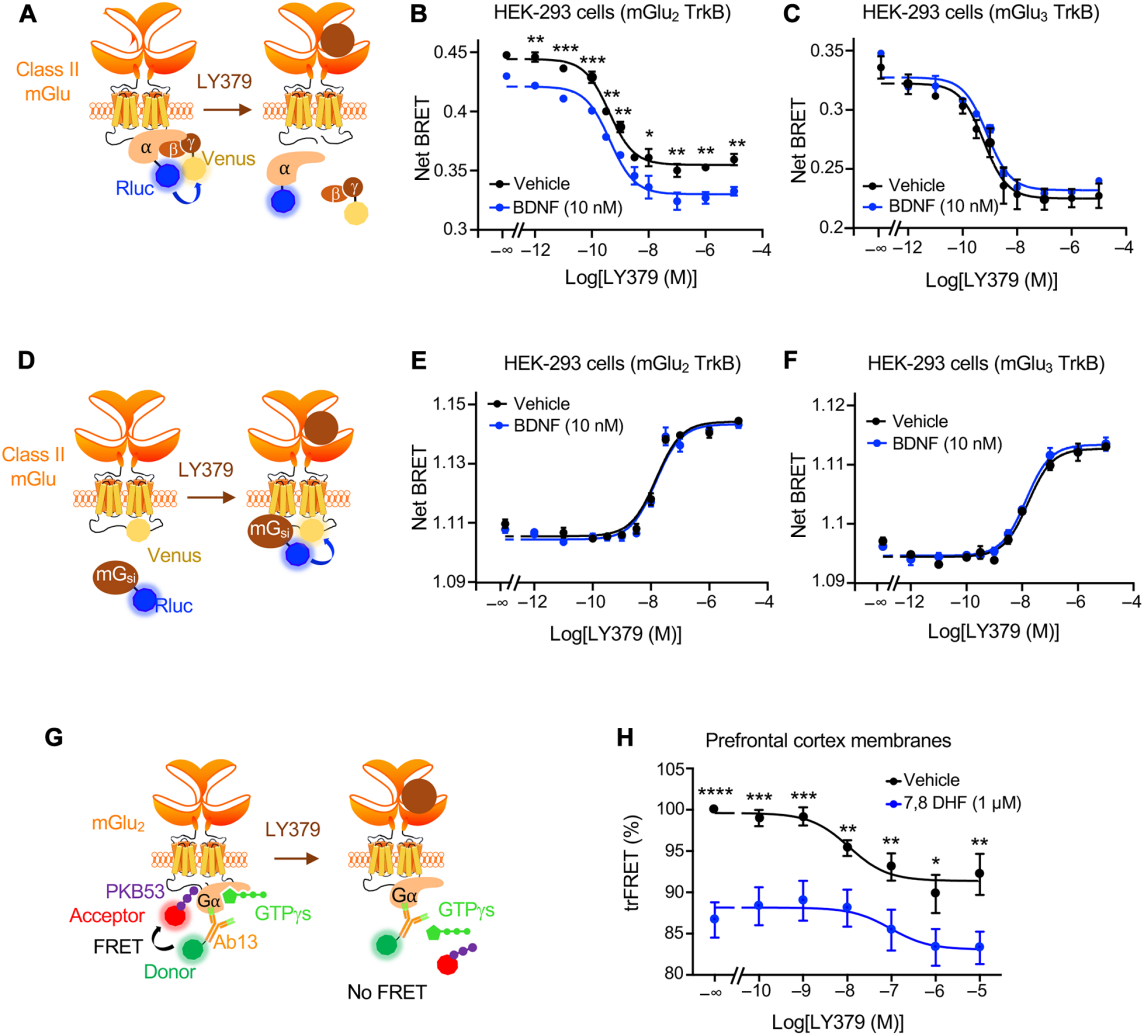
TrkB activation promotes mGlu_2_-operated G_i/o_ activation. (**A**) Schema of the BRET assay assessing Gα_o_ and Gβγ dissociation. (**B** and **C**) Gα_o_ and Gβγ dissociation induced by increasing concentrations of LY379268 in the presence or absence of 10 nM BDNF in HEK-293 cells expressing Gα_oA_-Rluc and Gγ-Venus subunits, human TrkB, and either human Flag-tagged mGlu_2_ (B) or mGlu_3_ (C). Curves show means ± SEM of net BRET ratio in three technical replicates of one experiment representative of three independent experiments performed in different sets of cultured cells. Two-way ANOVA (post hoc Šidák’s multiple comparison test): *P < 0.05, **P < 0.005, and ***P < 0.001: mGlu_2_/TrkB *F*_10,38_ = 163.4 and mGlu_3_/TrkB *F*_10,44_ = 68.47. (**D**) Schema of the BRET assay assessing G protein recruitment by mGlu_2_. (**E** and **F**) Representative BRET assay of miniGα_i_ recruitment by mGlu_2_ or mGlu_3_ induced by increasing concentrations of LY379268 in the presence or absence of BDNF in HEK-293 cells coexpressing miniGα_i_-Rluc, TrkB, and either human mGlu_2_-Venus (E) or mGlu_3_-Venus (F), respectively. Two-way ANOVA (post hoc Šidák’s multiple comparison test): mGlu_2_/TrkB *F*_11,48_ = 293.5 and mGlu_3_/TrkB *F*_10,22_ = 286.6. (**G**) Schema of the trFRET assay between PKB53 peptide, which specifically binds to the GTP-γ-S–bound active conformation of Gα_i_ proteins and an Ab13 antibody recognizing total Gα_i1,3_ protein. Upon agonist stimulation of GPCRs such as mGlu_2_, the guanine nucleotide exchange activity of the receptor promotes the release of GTP-γ-S from activated Gα_i_, leading to a decrease in trFRET. (**H**) Effect of exposure of membrane preparations of mice PFC to increasing concentrations of LY379268 after pretreatment with vehicle or 1 μM 7,8 DHF on Gα_i1,3_ activation. The data are representative of three independent experiments performed in triplicate on different membrane preparations. *P < 0.05, **P < 0.005, ***P < 0.001, and ****P < 0.0001 versus corresponding condition with 7,8-DHF *F*_6,109_ = 4862.

We next examined whether TrkB stimulation reciprocally affects mGlu_2_ signal transduction, by measuring mGlu_2_-operated dissociation of Gα subunit from Gβγ subunits using a BRET assay in HEK-293 cells (Fig. 5A). As expected, exposing cells to increasing concentrations of LY379268 induced the dissociation of the G_o_ protein in cells expressing mGlu_2_ (fig. S7A) or mGlu_3_ alone (fig. S7B), and in cells coexpressing mGlu_2_ and TrkB (Fig. 5B) or mGlu_3_ and TrkB (Fig. 5C). LY379268 did not induce the dissociation of G_o_ protein in cells expressing TrkB alone (fig. S7C). Treatment of cells expressing mGlu_2_ alone (fig. S7A) or mGlu_3_ alone (fig. S7B) or TrkB alone (fig. S7C) with BDNF (10 nM) had no effect on G_o_ protein dissociation. However, BDNF exposure increased G_o_ protein dissociation in cells coexpressing mGlu_2_ and TrkB (Fig. 5B). Similarly, BDNF exposure enhanced the responses induced by increasing concentrations of LY379268, without altering mGlu_2_ potency for LY379268 (pIC_50_: 9.10 ± 0.20 and 9.44 ± 0.06, *n* = 3, in cells exposed or not with BDNF, respectively, *P* = 0.986) (Fig. 5B). In contrast, BDNF treatment had no effect in cells coexpressing mGlu_3_ and TrkB (Fig. 5C), indicating that TrkB stimulation specifically enhances mGlu_2_-mediated but not mGlu_3_-mediated G_o_ signaling.

We next examined the impact of TrkB stimulation on the recruitment of G_i_ protein to mGlu_2_ by measuring the BRET signals between C-terminally Venus-tagged mGlu_2_ or mGlu_3_ and a chimeric Rluc8-tagged miniGα_si_ protein (Fig. 5D) (*23*). As expected, treating cells with LY379268 induced a recruitment of the miniG protein directly to the mGlu receptor in cells expressing mGlu_2_ (fig. S7D) or mGlu_3_ alone (fig. S7E) and cells coexpressing mGlu_2_ or mGlu_3_ and TrkB receptor (Fig. 5, E and F). G protein recruitment elicited by LY379268 was unaffected by BDNF cotreatment even in cells coexpressing TrkB and mGlu_2_ (Fig. 5E) or mGlu_3_ (Fig. 5F). Collectively, these results suggest that TrkB stimulation does not affect G protein recruitment to mGlu_2_ but stabilizes mGlu_2_ in an active conformation that promotes G protein activation in the absence or presence of agonist.

To further confirm these findings in an authentic tissue context, we performed a time-resolved Förster resonance energy transfer (trFRET)–based guanosine 5′-*O*-(3′-thiotriphosphate) (GTP-γ-S) binding assay in PFC membrane preparations. This assay relies on trFRET-based conformational sensors that recognize the active GTP-γ-S–bound state of endogenous Gα_i_ proteins and any conformation of Gα_i_ proteins, respectively (Fig. 5G). Upon agonist stimulation of GPCRs, the guanine nucleotide exchange activity of the receptor promotes the release of GTP-γ-S from activated Gα_i_ proteins, leading to a decrease in trFRET. Incubation of membranes with LY379268 induced a concentration-dependent decrease in the trFRET signal, indicative of nucleotide release from Gα_i_ proteins induced by the agonist-bound mGlu_2_ receptor (Fig. 5H). Pretreatment of membranes with 1 μM 7,8 DHF enhanced both basal and LY379268-induced response (Fig. 5H). These findings are reminiscent of our observations in HEK-293 cells showing that TrkB activation enhances mGlu_2_-mediated G protein activation. Together with data indicating that mGlu_2_ activation transactivates TrkB in mouse cortical neurons and PFC, they demonstrate a reciprocal cross-talk between native mGlu_2_ and TrkB.

### TrkB pharmacological inhibition prevents behavioral deficit alleviation by glutamatergic antipsychotics in mice treated subchronically with PCP

**Fig. 6.**
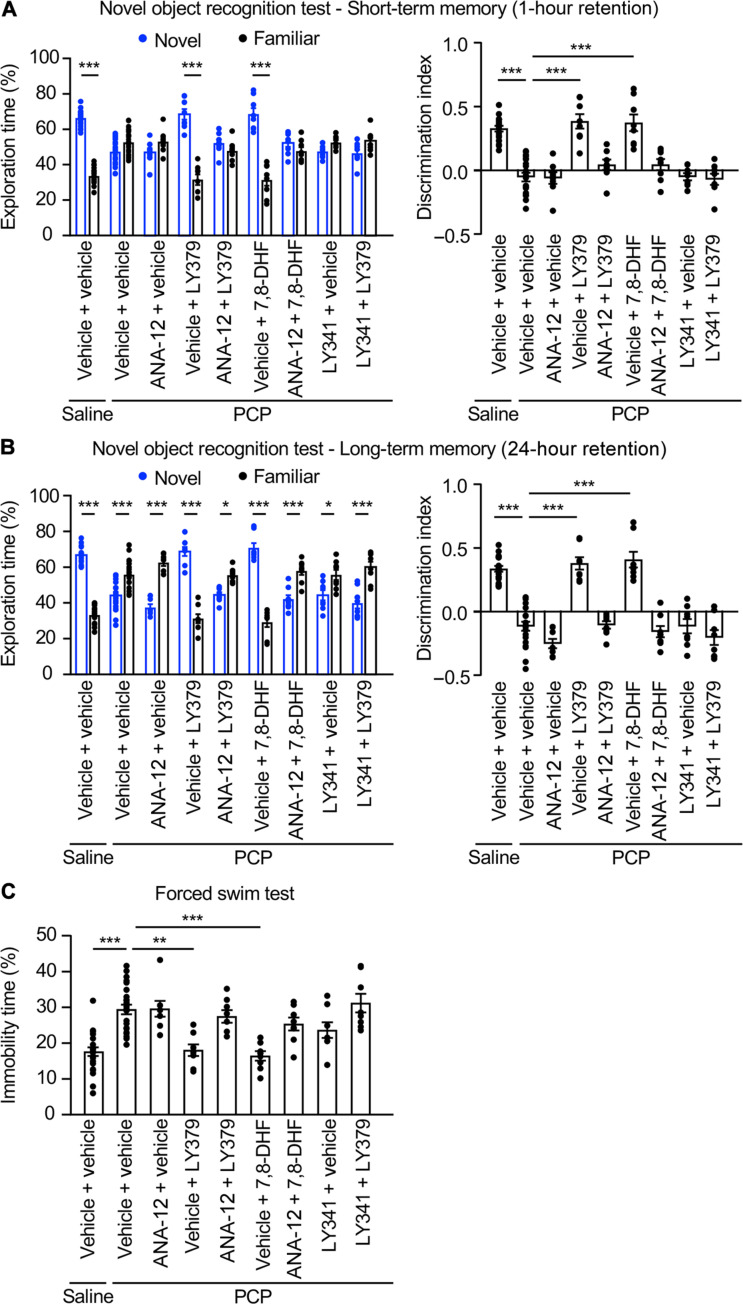
TrkB transactivation mediates rescue by a mGlu_2_ agonist of behavioral deficits in PCP-treated mice. (**A** and **B**) Mice injected with either saline or PCP as described in Materials and Methods were pretreated with either vehicle or ANA-12 (2.5 mg/kg) or LY341495 (3 mg/kg; LY341) and then injected with either vehicle or LY379268 (10 mg/kg; LY379) or 7,8-DHF (5 mg/kg). The left histograms represent the means ± SEM of the percentage of exploration time of novel versus familiar object. The right histograms represent the means ± SEM of the discrimination index. Each group included eight or more mice. Kruskal-Wallis (multiple comparisons compared to PCP vehicle, post hoc Dunn’s test): *P < 0.05 and ***P < 0.001. (**C**) Mice were treated as in (A) and (B). The histogram represents the percentage of immobility during the FST. Kruskal-Wallis test (multiple comparisons compared to PCP vehicle, post hoc Dunn’s test): **P < 0.01 and ***P < 0.001.

Mice subchronically treated with PCP (10 mg/kg, s.c. injection for five consecutive days during 2 weeks) (*24*) were used to explore the role of TrkB in the behavioral response to mGlu_2_ stimulation. Consistent with previous studies (*24*), PCP-treated mice showed deficits in short-term (Fig. 6A) and long-term (Fig. 6B) memory in the novel object recognition task. Acute administration of LY379268 (10 mg/kg, 30 min, i.p.) or 7,8-DHF (5 mg/kg, 1 hour, i.p.) before the test restored normal short- and long-term novelty discrimination in PCP-treated mice to levels similar to those observed in vehicle-treated mice. LY341495 (3 mg/kg, 30 min, i.p.) or ANA12 (2.5 mg/kg, 2 hours, i.p.) did not affect the memory capacities of PCP-treated mice (Fig. 6, A and B) when they were injected alone but prevented the alleviation of memory deficits elicited by LY379268 and 7,8-DHF (Fig. 6, A and B), respectively, in PCP-treated mice. The ability of LY379268 to alleviate short-term and long-term memory deficits in PCP-treated mice was also prevented by the administration of ANA-12, indicating a key influence of TrkB activation in the procognitive effect of the mGlu_2_ agonist. Neither LY341495 nor ANA-12 nor LY379268 (alone or in combination with LY341495 or ANA-12) nor 7,8-DHF (alone or in combination with ANA-12) altered short-term (fig. S8A) or long-term (fig. S8B) memory performance of vehicle-treated mice. None of the treatments significantly affected locomotor activity of mice (fig. S9).

We next examined the role of mGlu_2_/TrkB stimulation on immobility in the forced swim test (FST). This test is classically used as a model of depression and despair to screen for antidepressants, which decrease immobility time upon acute administration. Subchronic administration of NMDA receptor antagonists induce an increase in immobility time in the FST that is not affected by imipramine and likely does not reflect a depressive-like state (*25*, *26*). However, it is prevented by administration of atypical antipsychotics and is thus considered as a model of the negative symptoms of schizophrenia, such as reduced motivation and affective flattening (*25*). As expected, LY379268 or 7,8-DHF administration decreased the immobility time of PCP-treated mice to a level similar to that measured in vehicle-treated mice (Fig. 6C) (*26*). Injection of LY341495 or ANA-12, which alone had no effect on the immobility time of PCP-treated mice, prevented the rescue of PCP-induced motivation deficits by LY379268 and 7,8-DHF, respectively, in these mice. Reminiscent to its procognitive effects in the novel object recognition task, ANA-12 administration also abolished the rescue of PCP-induced motivation deficits (Fig. 6C), arguing for a key role of TrkB activation in the motivational effects of the mGlu_2_ agonist in PCP-treated mice. Administration of LY341495 or ANA-12 or LY379268 (alone or in combination with LY341495 or ANA-12) or 7,8-DHF (alone or in combination with ANA-12) did not modify immobility time of vehicle-injected mice in the FST (fig. S8C).

## DISCUSSION

While antipsychotics currently prescribed in patients with schizophrenia mainly target dopaminergic and serotonergic systems, a large body of evidence supports the notion that hyperfunction of the glutamatergic system has a key influence in the pathogenesis of the disease (*27*). This provided the impetus for the development of a recent generation of antipsychotics that behave as agonists or positive allosteric modulators of mGlu_2_. However, the neuronal signaling pathways and molecular targets that underlie their antipsychotic effects remain poorly characterized. One important issue is the high sequence homology between mGlu_2_ and mGlu_3_ (*28*), which makes highly challenging the design of mGlu_2_-specific ligands and, consequently, the characterization of signaling mechanisms specifically engaged by mGlu_2_, but not by mGlu_3_.

Taking advantage of our recently developed selective mGlu_2_ nanobody (*14*), we provide a first unbiased characterization of mGlu_2_ interactome in mice PFC. Analysis of protein-protein interactions between the 149 candidate mGlu_2_ partners previously reported in public databases revealed that more than two-third of them are part of a unique protein complex. This indicates that the protocol used to purify mGlu_2_ partners did not affect protein-protein interactions and outlines the relevance of our AP-MS strategy to get a global picture of the mGlu_2_ interactome in vivo, including not only direct receptor partners but also proteins recruited via their association with intermediate partners. Further supporting the relevance of the results of our interactomic screen, it identified several proteins previously reported as mGlu_2_ partners. These include the α subunit of the G_i1_ protein (*GNAI1*), consistent with the canonical coupling of mGlu_2_ to G_i/o_ proteins (*11*). Group II (mGlu_3_) and group III (mGlu_4_, mGlu_7_, and mGlu_8_) mGlu receptors, but not group I mGlu receptors (mGlu_1_ and mGlu_5_), were also identified in the mGlu_2_ interactome. These findings suggest that mGlu_2_ is colocalized with those receptors in some PFC neuronal populations, consistent with previous fluorescence in situ hybridization and single-cell RNA sequencing experiments indicating that most mGlu subtypes, with the exception of mGlu_6_, are coexpressed in the majority of cortical neurons. They also suggest that mGlu_2_ can form heteromers with mGlu_3_, mGlu_4_, mGlu_7_, and mGlu_8_ in PFC, corroborating previous observations using a trFRET assay in transfected cells indicating that group II and III mGlu receptors can heteromerize, whereas neither group II nor group III receptors form heteromers with group I receptors (*16*). Among possible heteromeric combinations, mGlu2-3 and mGlu2-4 heteromers have already been detected in mouse brain, especially the PFC (*29*, *30*). Furthermore, it has recently been proposed that mGlu_2_ and mGlu_3_ have a higher propensity to form heteromers compared to other mGlu receptor pairs, corroborating our results indicating that mGlu_3_ is the most significantly enriched among mGlu receptors identified in the mGlu_2_ interactome (*30*). The existence of mGlu_2_/mGlu_7_ and mGlu_2_/mGlu_8_ heteromers remains to establish in PFC, as one cannot rule out indirect recruitment mGlu_7_ and mGlu_8_ via formation of oligomeric mGlu receptor assemblies or association with different intermediate partners.

The specific identification of group II and III mGlu receptors in the mGlu_2_ interactome also reflects their predominant localization in neurons: group II receptors, including mGlu_2_, and group III receptors are mostly found in presynaptic compartment, while group I mGlu receptors are almost exclusively post-synaptic (*19*). Nevertheless, there is compelling evidence indicating that a fraction of mGlu_2_ is also located post-synaptically, where it can associate with the serotonin 5-HT_2A_ receptor, another major target of antipsychotics (*31*). It has been proposed that post-synaptic mGlu_2_/5-HT_2A_ heteromers constitutes an important target of antipsychotics (*32*) and that normal nonpsychotic state and the ability of 5-HT_2A_ and mGlu_2_ ligands to alleviate psychotic symptoms depend on a homeostatic balance of G_i/o_- and G_q_-dependent signaling under the control of post-synaptic mGlu_2_/5-HT_2A_ heteromers (*33*, *34*). Analysis of functional annotations within the mGlu_2_ interactome revealed a predominant presynaptic localization of the mGlu_2_-associated complex and a strong enrichment in biological processes and molecular functions characteristic of presynaptic receptors. Consistent with the absence of 5-HT_2A_ in our interactome, this suggests that our interactomic screen mostly identified the interactome of presynaptic mGlu_2_, while the interactome of post-synaptic receptor could not be identified, presumably due its lower abundance.

Our interactomic screen also identified subunits 2A and 2B of glutamate ionotropic receptor of *N*-methyl-d-aspartate types (*GRIN2A* and *GRIN2B*) that are expressed not only at the post-synapse as initially thought but also in the presynaptic compartment where they play a key role in shaping synaptic transmission and plasticity (*35*). A study has shown that activation of group II mGlu receptors promotes long-term potentiation induction at Schaffer collateral-CA1 pyramidal cell synapses through a mechanism involving enhancement of NMDA receptor–mediated currents, suggesting a functional cross-talk between mGlu_2/3_ and NMDA receptors (*36*). The importance of physical interaction of NMDA receptor subunits with mGlu_2_ and/or mGlu_3_ in modulation of synaptic plasticity remains to be established. Likewise, given the key influence of NMDA receptor hypofunction in the pathophysiology of schizophrenia (*37*), the association GRIN2A and GRIN2B with presynaptic mGlu_2_ and mGlu_3_ in the context of schizophrenia certainly warrants further exploration.

Among the newly identified mGlu_2_-interacting proteins, we focused on the RTK TrkB that displayed a robust and reproducible enrichment in the mGlu_2_ interactome, in view of (i) the decreased expression of TrkB and its natural ligand BDNF in patients with schizophrenia in comparison to general population (*15*), (ii) the association of BDNF Val^66^Met polymorphism that alters BDNF secretion and schizophrenia (*38*), and (iii) the ability of a specific TrkB agonist to reverse cognitive and synaptic plasticity deficits in a rat model of schizophrenia (*39*). Using PLA, we confirmed the association of TrkB with endogenous mGlu_2_ in various brain regions including PFC, OB, DG, and CB, whereas TrkB did not interact with the other group II member mGlu_3_. This result was confirmed by BRET experiments, which also suggest that TrkB directly binds to mGlu_2_. Furthermore, TrkB-mGlu_2_ interaction was promoted by agonist stimulation of either mGlu_2_ or TrkB and inhibited by a mGlu_2_ antagonist, indicating that it dynamically regulated by the conformational state of both receptors. This agonist-dependent physical complex formations between GPCRs and RTKs had only been demonstrated for a few receptor pairs (*22*), including AT1 angiotensin/epidermal growth factor receptor (*40*) and β_2_-adrenergic/vascular endothelial growth factor receptor 2 (*41*) and never for a class C GPCR.

These observations prompted further studies exploring the reciprocal influence of both receptors on their signal transduction, which revealed that mGlu_2_ stimulation induces TrkB activation, as assessed by its phosphorylation on Tyr^816^, in PFC neurons. Notably, TrkB phosphorylation level elicited by mGlu_2_ activation was comparable to that induced by direct TrkB stimulation by an agonist, suggesting that it is physiologically relevant. Reciprocally, TrkB stimulation promoted G_o_ activation upon coexpression of mGlu_2_ in the absence or presence of a mGlu_2_ agonist, indicating that TrkB can transactivate unliganded mGlu_2_. Transactivation of RTKs by GPCRs has been demonstrated for numerous GPCR/RTK pairs (*22*). For instance, previous studies have shown that TrkB is transactivated by adenosine A_2A_ receptor (*42*) and pituitary adenylate cyclase activating polypeptide (PACAP) receptor (*43*), a process underlying neurotrophic effects of adenosine and PACAP, while stimulation of mGlu_2_ transactivates insulin-like growth factor 1 receptor (*44*). Although less extensively observed, RTK ligands can also transactivate GPCRs, indicating that transactivation can be bidirectional (*22*, *45*). To our knowledge, the present study provides the first example of reciprocal transactivation between a GPCR and a RTK. Several GPCR/RTK transactivation mechanisms have been described depending on receptor pairings and cell types. They can be ligand dependent and result from ligand release or cleavage of a proligand by a protease, as well as ligand binding to their cognate receptor. Such a mechanism was not involved in TrkB transactivation induced by LY379268, as it was not prevented by a BDNF neutralizing antibody that inhibited TrkB activation induced by exogenous BDNF. Bidirectional GPCR/RTK transactivation can also result from the production of second messengers and activation of protein kinases upon activation of one of the receptors, which, in turn, promote phosphorylation on Ser/Thr or Tyr residues and activation of the unliganded partner receptor. Last, it can involve formation of RTK/GPCR heterocomplexes, allowing complex allosteric regulations of both receptors (*22*, *45*). BRET experiments indicating a direct interaction between TrkB and mGlu_2_ suggest that both receptors might behave as reciprocal positive allosteric modulators able to stabilize the partner receptor in an active conformation even in the absence of mGlu_2_ agonist. Notably, TrkB transactivation by mGlu_2_ also requires activation of Gα_i/o_ protein, since it was prevented by pretreatment of primary neurons with PTX. This suggests that additional partner proteins within the mGlu_2_/TrkB interactome might also contribute to the reciprocal transactivation of both receptors.

BDNF-TrkB signaling has long been considered a key player of neuronal development, synaptogenesis, as well as Hebbian and homeostatic plasticity (*38*). Over the two past decades, it has also emerged as critical factor underlying the effects of various psychiatric therapies (*46*). Both aminergic and fast-acting antidepressants promote the release of BDNF, and activation of TrkB signaling underlies their antidepressant effects despite the distinct time course and mechanism of action of both categories of antidepressants (*47*). Intriguingly, antidepressant-like effects of mGlu_2_ antagonists in mouse models of depression seem to involve BDNF release (*48*, *49*), whereas we show in the present study that mGlu_2_ agonists transactivate TrkB in healthy mice through a distinct mechanism that is independent of BDNF release. Last, mood stabilizers such as lithium and valproic acid also increase BDNF production, and BDNF-TrkB signaling has been involved in the anti-manic effects of lithium (*46*). However, the role of BDNF-Trk signaling in the response to antipsychotics is much less documented. While typical antipsychotics generally reduced BDNF expression in the brain, atypical antipsychotics such as clozapine often increase BDNF expression (*50*). Furthermore, the effects of antipsychotics on BDNF/TrkB expression seem to depend on the brain region examined and the duration of the treatment (*38*, *51*). Although the influence of glutamatergic antipsychotics on BDNF release remains to be elucidated, we show in the present study a key role of TrkB activation in the ability of glutamatergic antipsychotics to alleviate cognitive and negative symptoms in mice treated subchronically with PCP, providing a first link between BDNF-TrkB signaling and therapeutic response to antipsychotics. As TrkB pharmacological inhibition affects both TrkB alone signaling and TrkB/mGlu_2_ heteromer signaling, a tool that specifically disrupts TrkB/mGlu_2_ interaction without affecting TrkB response to BDNF, would certainly provide valuable information about the relative contribution of direct TrkB/mGlu_2_ transactivation versus more indirect signaling cross-talk mechanisms in the behavioral response to glutamatergic antipsychotics. The ability of BDNF-TrkB signaling to sustain or enhance neuroplasticity altered in schizophrenia might explain the improved therapeutic efficacy of glutamatergic antipsychotics compared to currently available monoaminergic antipsychotics in alleviating negative and cognitive symptoms of schizophrenia.

## MATERIALS AND METHODS

### Reagents

LY379268 disodium salt (#5064), LY341495 disodium salt (#5062), ANA12 (#4781), and BDNF (#2837) were from Tocris. 7,8-DHF (#D5446) and PCP hydrochloride (#P3029) were from Sigma-Aldrich.

### Antibodies

The rabbit anti-TrkB antibody (#13129-1-AP) was from Proteintech. The rabbit anti–p–Tyr^816^-TrkB antibody (#ABN1381) and the chicken anti–glyceraldehyde-3-phosphate dehydrogenase (GAPDH) antibody (#ab2302) were from Merck Millipore. The mouse anti-mGlu_2_ (#ab106811) was from Abcam. The mouse anti-Flag antibody (#F3165) was from Sigma-Aldrich. The anti-rabbit horseradish peroxidase (HRP)–conjugated antibody (GENA934V) and the anti-mouse HRP-conjugated antibody (GENA931V) were from Sigma-Aldrich. The anti-chicken HRP-conjugated antibody (ab7118) was from Abcam. The Alexa Fluor 488–conjugated anti-rabbit antibody was from Invitrogen (A11034). The llama anti-mGlu_2_ nanobody with C-terminal polyHis tag was a DN1 version produced in-house (*14*). The rabbit anti–p–Y^783^-PLCγ1 antibody was from Cell Signaling Technology.

### Cell cultures

HEK-293 N-type cells were from the American Type Culture Collection (ATCC) (Anassas, VI, ATCC CRL-1573). All experiments were performed using a previously characterized batch of cells (isolate #3) (*52*). Cells were grown in Dulbecco’s modified Eagle’s medium (DMEM) + GlutaMAX (Thermo Fisher Scientific, reference no. 10566016) supplemented with 10% heat-inactivated fetal bovine serum (Thermo Fisher Scientific, reference no. 10099-133) and penicillin (100 U/ml)-streptomycin (0.1 mg/ml) (Thermo Fisher Scientific, reference no. 15140-122) and maintained in humidified atmosphere containing 5% CO_2_ at 37°C. Cells were passed twice a week and used between passages 10 and 20. The absence of mycoplasma was assessed every month using the MycoAlert Mycoplasma Detection Kit (Lonza, reference no. LT07-118). Cells were transfected in suspension using polyethylenimine (Polyscience, reference no. 24765) and used 2 days after transfection for all experiments. Mock cells were transfected with empty plasmids.

Primary cultures of cortical neurons were prepared from 16.5-day-old embryonic mice, as previously described (*53*). All treatments were performed after 14 days in culture [days in vitro (DIV14)].

### Animals

Male wild-type C57BL/6J mice were purchased from Janvier Laboratories. Male *GRM2* KO mice were obtained from the RIKEN Institute (RBRC01351 B6.129S-*Grm2* < tm1Nak>/NakRbrc). Mice were housed under standardized conditions with a 12-hour light/12-hour dark cycle, 22°C temperature, 55% humidity, and free access to food and water. Biochemistry and immunohistochemistry experiments were conducted at post-natal day 60. Animal husbandry and experimental procedures were performed in compliance with the animal use and care guidelines of the French Agriculture Ministry, European ethics standards (86/609/EEC), and decrees of the French national ethics committee (B34-172-41). Drugs were injected to mice (~25 g) intraperitoneally at LY379268 (10 ml/kg) and LY341495 were dissolved in 5% dimethyl sulfoxide (DMSO), 5% Tween 80, and 0.9% NaCl. ANA-12 was dissolved in 10% DMSO, 5% Tween 80, and 0.9% NaCl. 7,8-DHF was dissolved in 17% DMSO, 5% Tween 80, and 0.9% NaCl. Mice were injected with either vehicle or 7,8-DHF (5 mg/kg, 1 hour) or LY379268 (10 mg/kg, 30 min). ANA12 (2.5 mg/kg) and LY341495 (3 mg/kg) were injected 2 hours and 15 min before agonist administration, respectively. For behavioral tests, mice were subcutaneously injected with either vehicle or PCP (10 mg/kg) in a solution of 0.9% NaCl at 10 ml/kg daily for 10 days (once daily, on days 1 to 5 and days 8 to 12), as previously described (*24*). Behavioral tests were performed 2 days after the last PCP injection (post-natal day 72).

### AP and MS analysis of endogenous mGlu_2_ interactome

Prefrontal cortices were rapidly dissected (coronal section from Bregma 3.56 to 2 mm) and homogenized in an ice-cold lysis buffer containing lauryl maltose neopentyl glycol 3 (2%; Anatrace, #NG310), sodium chloride (150 mM), tris-HCl (50 mM; pH 7.4), EDTA (5 mM), EGTA (2 mM), sodium orthovanadate (1 mM), sodium fluoride (50 mM), sodium ß-glycerophosphate (25 mM), sodium pyrophosphate (5 mM), and cOmplete protease inhibitor EDTA free (1 pellet for 20 ml; Roche Diagnostics, #45148300). Lysates were centrifuged (10 min, 16,000*g*) to eliminate insoluble material. Soluble proteins were quantified by bicinchoninic acid assay (Merck, reference nos. B29643 and C2284) and equal protein amounts (1 mg) were incubated with anti-mGlu_2_ nanobody (5 μg) and nickel–nitrilotriacetic acid agarose beads (QIAGEN, reference no. 30210). Control immunoprecipitations were performed with a nonimmune nanobody. Experiments were performed in triplicate, each from the PFC of one mouse. Samples were centrifuged (5 min, 1000*g*) and washed three times with lysis buffer containing 500 mM NaCl and once with lysis buffer containing 150 mM NaCl, and immunoprecipitated proteins were eluted with lysis buffer supplemented with imizadole (250 mM). After reduction (dithiothreitol, 10 mM, 20 min at 25°C) and alkylation (iodoacetamide 50 mM, 30 min at 25°C) of cysteines, affinity-retained proteins were resolved by SDS–polyacrylamide gel electrophoresis and stained with colloidal Coomassie Blue (Euromedex, #10-0911). Gel lanes were cut into six bands, destained in 30% acetonitrile (ACN), 50 mM TEABC [triethylammonium bicarbonate (pH 8.0)], and digested in-gel with trypsin (10 ng/ml; Gold, Promega) overnight at 25°C in 50 mM TEABC. Peptides were eluted with two baths of 30% ACN and 50 mM TEABC and two baths of 30% ACN and 2.5% formic acid. Peptides were dried in a SpeedVac, resuspended in 2% ACN and 0.1% formic acid and analyzed on a Q Exactive Plus mass spectrometer coupled to an Ultimate 3000 nanoHPLC (Thermo Fisher Scientific). Desalting and preconcentration of samples were performed online on a PepMap precolumn (0.3 mm by 10 mm; Dionex) in buffer A (2% acetonitrile and 0.1% formic acid). A gradient consisting of 2 to 40% buffer B (B = 99.9% acetonitrile with 0.1% formic acid; 3 to 33 min), 40 to 80% B (33 to 34 min), 80 to 0% B (49–50 min) and equilibrated for 20 min in 0% B (50 to 70 min) was used to elute peptides at 300 nl/min from a PepMap capillary reverse-phase column (0.075 mm by 150 mm; Thermo Fisher Scientific). Mass spectra were acquired using a top 10 Higher-energy collisional dissociation data-dependent acquisition method. The mass spectrometer was programmed to perform a full scan in the Orbitrap analyzer (60,000 resolution, target ion numbers of 500,000, and Th mass range of 375 to 1500) with the top 10 ions from each scan selected for MS/MS (isolation window 1.2 mass/charge ratio, 30,000 resolution). Fourier transform (FT) spectra were internally calibrated using a single lock mass (445.1200 Th). Data were searched against the canonical *Mus musculus* Complete Proteome Set database downloaded on 14 January 2019 (www.uniprot.org) using MaxQuant (v 1.6.3.4; www.maxquant.org) with standard parameters: cysteine carbamidomethylation as a fixed modification, methionine oxidation and protein N-terminal acetyl as variable modifications, 7–parts per million precursor mass tolerance, 0.02-Da fragment mass tolerance, and trypsin/P digestion. Proteins were identified with at least two unique/razor peptides with 1% FDR (decoy database). Protein MS intensities were extracted using Skyline software (v 19.1; https://skyline.ms), considering only unique peptides. Protein intensities were normalized to the total protein intensities in each biological sample. The significance of each protein enrichment was calculated with a Student’s *t* test (one-sided test, correction s0 = 0).

The list of proteins exhibiting a significant enrichment in mGlu_2_ immunoprecipitates, compared with control immunoprecipitates (*P* value < 0.05), was used as input in String software (https://string-db.org) for representation of protein-protein physical interactions previously reported with score of ≥0.4 in String databases. The list of proteins of the most connected network was uploaded in g:Profiler software (https://biit.cs.ut.ee/gprofiler/gost) to calculate enrichment in GO functional annotations against the whole list of proteins identifed in our experiments.

### Immunoprecipitation from transfected HEK-293 cells and primary neurons

HEK-293 cells were transfected with plasmids encoding human Flag-mGlu_2_ or human Flag-mGlu_3_ in combination or not of a plasmid encoding human TrkB or empty vector at 40 to 50% confluence using Lipofectamine 2000 (Invitrogen). Forty-eight hours after transfection, cells were starved of serum for 4 hours and lysed in the same ice-cold lysis buffer as for the AP protocol. The immunoprecipitation was performed with as previously described (*54*). Briefly, solubilized proteins (1.5 mg) were incubated with anti-Flag M2 affinity gel beads (Sigma-Aldrich, #A2220) for 4 hours at 4°C. Samples were centrifuged (5 min, 1000*g*) and washed three times with an ice-cold solution of 500 mM NaCl and phosphatase inhibitors and once with 150 mM NaCl and phosphatase inhibitors. Immunoprecipitated proteins were then eluted in Laemmli sample buffer.

Immunoprecipitation from primary cultured cortical neurons was performed using 0.5 mg of solubilized neuronal proteins and the mGlu_2_ nanobody (2 μg) as described for the AP-MS procedure. Control immunoprecipitations were performed with a nonimmune nanobody. Samples were centrifuged (5 min, 1000*g*) and washed three times with an ice-cold solution of 500 mM NaCl and phosphatase inhibitors and once with 150 mM NaCl and phosphatase inhibitors. Immunoprecipitated proteins were then eluted in Laemmli sample buffer.

### Western blotting

Proteins were resolved by SDS polyacrylamide (10%) gel electrophoresis and transferred electrophoretically (Trans-Blot Turbo, Bio-Rad) to nitrocellulose membrane (Bio-Rad, #1620113). Membranes were saturated with tris-buffered saline supplemented with 0.1% Tween 20, 3% skimmed milk, and 3% bovine serum albumin (BSA) and incubated with primary antibodies (anti–p–Tyr^816^-TrkB, 1:2000; anti-TrkB, 1:1000; anti-Flag, 1:2000; anti-mGlu_2_, 1:2000; anti-GAPDH, 1:5000, anti–p–Y^783^-PLCγ1, 1:2000) and then with anti-rabbit, anti-mouse, or anti-chicken HRP-conjugated secondary antibodies (1:5000). Immunoreactivity was detected with an enhanced chemiluminescence method (Western lightning Plus-ECL, PerkinElmer) using a ChemiDoc MP Imaging System (Bio-Rad). Immunoreactive bands were quantified by densitometry using the Image Lab software (v.6.0.1). In TrkB phosphorylation analyses, the signal of p–Y^816^-TrkB was normalized to the signal of total TrkB detected in the sample.

### Bioluminescence resonance energy transfer

For saturation assay, cells were cotransfected with a constant amount of constructs encoding TrkB-Rluc or mGlu_5_-Rluc (20 ng per 100,000 cells, BRET donors) and increasing amounts of constructs encoding mGlu_2_-Venus or mGlu_3_-Venus or mGlu_2_C3-Venus or membrane bound CAAX-Venus or cytoplasmic Venus (from 0 to 40 ng per 100,000 cells, BRET acceptors). For G protein dissociation assay, cells were cotransfected with constructs encoding Gα_o_-Rluc (10 ng per 100,000 cells), Flag-Gβ (20 ng per 100,000 cells), and Gγ-Venus (20 ng per 100,000 cells) and constructs encoding human Flag-mGlu_2_ (20 ng per 100,000 cells) or Flag-mGlu_3_ (20 ng per 100,000 cells) in combination or not with a plasmid encoding human TrkB (20 ng per 100,000 cells), as indicated. For miniG protein recruitment assay, cells were cotransfected with construct encoding miniG_si_-Rluc chimeric protein (10 ng per 100,000 cells) and constructs encoding mGlu_2_-Venus or mGlu_3_-Venus (20 ng per 100,000 cells) in combination with a plasmid encoding human TrkB (20 ng per 100,000 cells), as indicated. In all experiments, cells were cotransfected with a plasmid encoding the plasma membrane glutamate transporter EAAC1 (6 ng per 100,000 cells) to maintain extracellular glutamate concentration at low levels and minimize mGlu receptor activation by endogenously released glutamate. Forty-eight hours after transfection, cells were starved of serum for 1 hour with DMEM + GlutaMAX and incubated for 10 min with coelanterazine H (5 μM final concentration) in Dulbecco’s phosphate-buffered saline (PBS) (Thermo Fisher Scientific, reference no. 14040083). For G protein dissociation and miniG recruitment assays, cells were stimulated for 10 min with increasing concentrations of LY379268 (10^−12^ to 10^−5^ M) in the presence or absence of 10 nM BDNF. BRET signals were measured using a Mithras LB940 instrument. Rluc8 emission was measured at 410 ± 80 nm, and Venus emission was measured at 515 ± 30 nm. Net BRET ratios were calculated as previously described (*55*) with the formula net BRET ratio = ratio [(Venus emission)/(Rluc8 emission)] in tested well − ratio [(Venus emission)/(Rluc8 emission)] in Rluc8 alone condition.

### p–Y^816^-TrkB immunolabeling

For analysis of TrkB phosphorylation in primary cultures, cortical neurons cultured on glass coverslips were fixed in 4% paraformadehyde in PBS, permeabilized in PBS solution containing 5% BSA and 0.1% Triton X-100 for 30 min, and incubated with the anti–p–Y^816^-TrkB antibody (1:1000) overnight at 4°C in PBS containing 5% BSA and 0.1% Triton X-100. After three washes in PBS, cells were incubated in PBS containing 5% BSA and 0.1% Triton X-100 for 1 hour with an Alexa Fluor 488–conjugated anti-rabbit antibody (1:1000). After three washes in PBS, cells were incubated with DAPI (4 μM) for 5 min in PBS and washed again three times with PBS. Coverslips were mounted on superfrost ultra plus slides (Thermo Fisher Scientific, reference no. 10417002) using fluorescent mounting medium (Dako, reference no. S3023). Immunofluorescence staining was imaged using an Axio Imager Z1 microscope equipped with apotome (Carl Zeiss). Image analysis was performed with Fiji software (v2.1.0), and results are expressed as the number of anti–p–Y^816^-TrkB–positive pixels per field (RawIntDen).

For analysis of TrkB phosphorylation in mouse PFC, mice were injected with 150 μl of Euthasol Vet (33 mg/kg; diluted in 0.9% NaCl, TVM Laboratory Innovative Animal Health). Five min after injection, mice were intracardially perfused with a fixing solution containing 4% paraformaldehyde, PBS (pH 7.5), 10 mM sodium fluoride, and 2 mM sodium orthovanadate. Brains were post-fixed for 48 hours in the same solution and successively transferred to PBS containing 10, 20, and, lastly, 30% sucrose. Coronal slices (30-μm thick) were cut with a Vibratome (Leica). Epitopes were first unmasked for 30 min with 1% sodium borohydride in PBS. After three washes, slices were permeabilized in PBS solution containing 5% goat serum, 5% horse serum, and 0.1% Triton X-100 for 2 hours, as previously described (*56*). Slices were then incubated with the anti–p–Y^816^-TrkB antibody (1:500) for 48 hours at 4°C. After three washes in PBS, they were incubated with an Alexa Fluor 488–conjugated anti-rabbit antibody (1:500) for 90 min in PBS solution containing 5% goat serum, 5% horse serum, and 0.1% Triton X-100. After three washes, slices were incubated 5 min in PBS with DAPI (4 μM) and washed again three times. Slices were mounted on superfrost ultra plus slides (Thermo Fisher Scientific, reference no. 10417002) using fluorescent mounting medium (Dako, reference no. S3023). Immunofluorescence staining was imaged using a TCS SP8 X confocal microscope (Leica). Image analysis was performed using Fiji software (v2.1.0), and results are expressed as the number of anti–p–Y^816^-TrkB–positive pixels per field (RawIntDen).

### Immunolabeling with anti-mGlu_2_ nanobody

Prefrontal cortex coronal sections were incubated for 1 hour at 20°C with a blocking solution (3% BSA and 0.1% Triton X-100 in PBS). Sections were then incubated with d2-labeled anti-mGlu_2_ nanobody (200 nM in the blocking solution) overnight at 4°C and were washed with PBS and distilled water. Fluoroshield with 4′,6-diamidino-2-phenylindole (DAPI) (Sigma-Aldrich) was used as mounting medium to preserve the fluorescence of brain sections. Images were obtained with a slide scanner Axio scan Z1 microscope (Carl Zeiss Microscopy) by performing full-section mosaics at ×20 magnification.

### Proximity ligation assay

For experiments performed in cell cultures, HEK-293 cells were incubated with anti-TrkB (1:1000) and anti-Flag (1:1000) antibodies overnight at 4°C. For experiments in mice brain, sagittal brains slices (16-μm thick) were cut with a cryostat (Leica). Slices were incubated with anti-TrkB (1:500), anti–6-His (1:500) antibodies, and anti-mGlu_2_ nanobody (200 nM) for 48 hours at 4°C. PLA labeling was then performed according to the manufacturer’s instructions (Duolink In Situ Detection Reagents Orange, Sigma-Aldrich). HEK-293 cells and brain slices were imaged on a confocal microscope (Leica TCS SP8 X). All acquisitions were made the same day under the same confocal laser power settings to minimize variability among samples. Quantitative analysis of PLA complexes was performed as previously described (*57*).

### trFRET assay for native G_i_ protein activation

Prefrontal cortices of 2-month-old mice were homogenized with a Dounce homogenizer in 1 ml of homogenization buffer containing 25 mM tris HCl (pH 7.4), 10 mM MgCl_2_, 0.5 mM EDTA, 10 mM MgCl_2_, 10% sucrose, and cOmplete protease inhibitor cocktail (Roche). Tissue debris were eliminated by a 10-min centrifugation at 1500*g*. Supernatants were centrifuged again for 15 min at 13,300*g* to pellet membranes. Membranes were then resuspended in 500 μl of homogenization buffer, snap-frozen, and stored at −80°C. The assay was performed in 386-well plates (ProxiPlate 384 Plus, PerkinElmer). All reagents used for the trFRET assay were diluted in trFRET Tag-lite buffer (Cisbio Bioassays Revvity). Before the assay, the biotinylated PKB53 peptide (50 nM; Cisbio Bioassays Revvity), which specifically binds to the active conformation of Gα_i_ proteins, which releases bound GTP-γ-S upon GPCR activation, was pre-incubated with 25 nM streptavidin-XL665 (trFRET acceptor, Cisbio Bioassays Revvity) for 30 min at 20°C. For the assay, 2.5 μg of PFC membranes was incubated for 10 min at 20°C with increasing concentrations of LY379268 in the presence or absence of 1 μM 7,8-DHF in the trFRET Tag-lite buffer supplemented with 1 nM GTP-γ-S. The XL665-PKB53 complex and a terbium-labeled Ab13 antibody (trFRET donor, 0.25 nM; Cisbio Bioassays Revvity) recognizing all conformations of Gα_i1,3_ proteins were then added for 3 hours at 20°C. The trFRET signal was measured using a PHERAstar microplate reader (BMG LABTECH) with the following setup: excitation at 337 nm (40 flashes per well), donor emission at 620 nm, acceptor emission at 665 nm, integration delay of 60 μs, and integration time of 400 μs.

### Behavioral studies

#### Object recognition test

This task was performed as previously described (*58*). On day 1, mice were habituated to the arena for 10 min. On day 2, they were subjected to a 5-min training session with two similar objects (dices or Lego plastic toys of approximately 3 cm in width and length and 5 cm in height), a 1-hour retention interval, and a 5-min test session in which one of the objects was replaced by a different object (short-term memory). On day 3, mice were subjected to another 5-min test session in which the previously replaced object was replaced again by a novel object (long-term memory). The experiments were video-recorded, and exploration times (nose in contact or sniffing at less than 1 cm) were measured double blinded. Discrimination indexes were calculated using the formula [(exploration time of novel object − exploration time of familiar object)/total object exploration time].

#### Forced swim test

FST was performed as previously described (*25*). Testing was carried out in a 2-liter glass beaker (10 cm in diameter, 30 cm in height) filled with 1.4 liter of tempered water. Mice were left to swim for a 5-min test session. The experiments were video-recorded, and the immobility time was measured double blinded. Percentage of immobility during the 5-min session test was compared between groups.

### Statistical analysis

Statistical analysis was performed using Prism (v. 8.0, GraphPad Software Inc). Histograms or curves show means ± SEMs. Normal distribution of values was evaluated by Shapiro-Wilk normality test. Biochemistry and immunocytochemistry experiments were analyzed by one-way analysis of variance (ANOVA) followed by Dunnett’s test. PLA experiments were analyzed by two-way ANOVA followed by either Dunnett’s test or Šidák’s multiple comparison test. Immunohistochemistry and BRET experiments were analyzed by two-way ANOVA followed by Šidák’s multiple comparison test. Behavioral experiments were analyzed by Kruskal-Wallis followed by Dunn’s multiple comparisons test. For each comparison, *F* value, number of independent biological replicates, and sample size (or number of animals per condition) are indicated in the corresponding figure legends.

## References

[1] M. J. Millan, A. Andrieux, G. Bartzokis, K. Cadenhead, P. Dazzan, P. Fusar-Poli, J. Gallinat, J. Giedd, D. R. Grayson, M. Heinrichs, R. Kahn, M.-O. Krebs, M. Leboyer, D. Lewis, O. Marin, P. Marin, A. Meyer-Lindenberg, P. McGorry, P. McGuire, M. J. Owen, P. Patterson, A. Sawa, M. Spedding, P. Uhlhaas, F. Vaccarino, C. Wahlestedt, D. Weinberger, Altering the course of schizophrenia: Progress and perspectives. Nat. Rev. Drug Discov. 15, 485–515 (2016). 26939910 10.1038/nrd.2016.28

[2] R. S. E. Keefe, R. M. Bilder, S. M. Davis, P. D. Harvey, B. W. Palmer, J. M. Gold, H. Y. Meltzer, M. F. Green, G. Capuano, T. S. Stroup, J. P. M. Evoy, M. S. Swartz, R. A. Rosenheck, D. O. Perkins, C. E. Davis, J. K. Hsiao, J. A. Lieberman, CATIE Investigators, Neurocognitive Working Group, Neurocognitive effects of antipsychotic medications in patients with chronic Schizophrenia in the CATIE trial. Arch. Gen. Psychiatry 64, 633–647 (2007). 17548746 10.1001/archpsyc.64.6.633

[3] S. T. Patil, L. Zhang, F. Martenyi, S. L. Lowe, K. A. Jackson, B. V. Andreev, A. S. Avedisova, L. M. Bardenstein, I. Y. Gurovich, M. A. Morozova, S. N. Mosolov, N. G. Neznanov, A. M. Reznik, A. B. Smulevich, V. A. Tochilov, B. G. Johnson, J. A. Monn, D. D. Schoepp, Activation of mGlu2/3 receptors as a new approach to treat schizophrenia: A randomized phase 2 clinical trial. Nat. Med. 13, 1102–1107 (2007). 17767166 10.1038/nm1632

[4] J. Maksymetz, S. P. Moran, P. J. Conn, Targeting metabotropic glutamate receptors for novel treatments of schizophrenia. Mol. Brain 10, 15 (2017). 28446243 10.1186/s13041-017-0293-z PMC5405554

[5] M. L. Woolley, D. J. Pemberton, S. Bate, C. Corti, D. N. C. Jones, The mGlu2 but not the mGlu3 receptor mediates the actions of the mGluR2/3 agonist, LY379268, in mouse models predictive of antipsychotic activity. Psychopharmacology 196, 431–440 (2008). 18057917 10.1007/s00213-007-0974-x

[6] M. J. Fell, K. A. Svensson, B. G. Johnson, D. D. Schoepp, Evidence for the role of metabotropic glutamate (mGlu)2 not mGlu3 receptors in the preclinical antipsychotic pharmacology of the mGlu2/3 receptor agonist (–)-(1*R*,4*S*,5*S*,6*S*)-4-amino-2-sulfonylbicyclo[3.1.0]hexane-4,6-dicarboxylic acid (LY404039). J. Pharmacol. Exp. Ther. 326, 209–217 (2008). 18424625 10.1124/jpet.108.136861

[7] S. Dogra, P. J. Conn, Metabotropic glutamate receptors as emerging targets for the treatment of schizophrenia. Mol. Pharmacol. 101, 275–285 (2022). 35246479 10.1124/molpharm.121.000460 PMC9092465

[8] B. Moghaddam, D. Javitt, From revolution to evolution: The glutamate hypothesis of schizophrenia and its implication for treatment. Neuropsychopharmacology 37, 4–15 (2012). 21956446 10.1038/npp.2011.181 PMC3238069

[9] B. J. Kinon, B. A. Millen, L. Zhang, D. L. McKinzie, Exploratory analysis for a targeted patient population responsive to the metabotropic glutamate 2/3 receptor agonist pomaglumetad methionil in schizophrenia. Biol. Psychiatry 78, 754–762 (2015). 25890643 10.1016/j.biopsych.2015.03.016

[10] M. de la Fuente Revenga, D. Ibi, T. Cuddy, R. Toneatti, M. Kurita, M. K. Ijaz, M. F. Miles, J. T. Wolstenholme, J. González-Maeso, Chronic clozapine treatment restrains via HDAC2 the performance of mGlu2 receptor agonism in a rodent model of antipsychotic activity. Neuropsychopharmacology 44, 443–454 (2019). 30038413 10.1038/s41386-018-0143-4 PMC6300555

[11] S. R. Ikeda, D. M. Lovinger, B. A. McCool, D. L. Lewis, Heterologous expression of metabotropic glutamate receptors in adult rat sympathetic neurons: Subtype-specific coupling to ion channels. Neuron 14, 1029–1038 (1995). 7538309 10.1016/0896-6273(95)90341-0

[12] P. Chavis, H. Shinozaki, J. Bockaert, L. Fagni, The metabotropic glutamate receptor types 2/3 inhibit L-type calcium channels via a pertussis toxin-sensitive G-protein in cultured cerebellar granule cells. J. Neurosci. 14, 7067–7076 (1994). 7965099 10.1523/JNEUROSCI.14-11-07067.1994 PMC6577284

[13] I. Feinberg, Schizophrenia: Caused by a fault in programmed synaptic elimination during adolescence? J. Psychiatr. Res. 17, 319–334 (1982). 7187776 10.1016/0022-3956(82)90038-3

[14] P. Scholler, D. Nevoltris, D. de Bundel, S. Bossi, D. Moreno-Delgado, X. Rovira, T. C. Møller, D. El Moustaine, M. Mathieu, E. Blanc, H. McLean, E. Dupuis, G. Mathis, E. Trinquet, H. Daniel, E. Valjent, D. Baty, P. Chames, P. Rondard, J.-P. Pin, Allosteric nanobodies uncover a role of hippocampal mGlu2 receptor homodimers in contextual fear consolidation. Nat. Commun. 8, 1967 (2017). 29213077 10.1038/s41467-017-01489-1 PMC5719040

[15] T. Hashimoto, S. E. Bergen, Q. L. Nguyen, B. Xu, L. M. Monteggia, J. N. Pierri, Z. Sun, A. R. Sampson, D. A. Lewis, Relationship of brain-derived neurotrophic factor and its receptor TrkB to altered inhibitory prefrontal circuitry in schizophrenia. J. Neurosci. 25, 372–383 (2005). 15647480 10.1523/JNEUROSCI.4035-04.2005 PMC6725470

[16] E. Doumazane, P. Scholler, J. M. Zwier, E. Trinquet, P. Rondard, J.-P. Pin, A new approach to analyze cell surface protein complexes reveals specific heterodimeric metabotropic glutamate receptors. FASEB J. 25, 66–77 (2011). 20826542 10.1096/fj.10-163147

[17] S. Lin, S. Han, X. Cai, Q. Tan, K. Zhou, D. Wang, X. Wang, J. Du, C. Yi, X. Chu, A. Dai, Y. Zhou, Y. Chen, Y. Zhou, H. Liu, J. Liu, D. Yang, M.-W. Wang, Q. Zhao, B. Wu, Structures of Gi-bound metabotropic glutamate receptors mGlu2 and mGlu4. Nature 594, 583–588 (2021). 34135510 10.1038/s41586-021-03495-2

[18] J. Reimand, R. Isserlin, V. Voisin, M. Kucera, C. Tannus-Lopes, A. Rostamianfar, L. Wadi, M. Meyer, J. Wong, C. Xu, D. Merico, G. D. Bader, Pathway enrichment analysis and visualization of omics data using g:Profiler, GSEA, Cytoscape and EnrichmentMap. Nat. Protoc. 14, 482–517 (2019). 30664679 10.1038/s41596-018-0103-9 PMC6607905

[19] A. Bodzęta, N. Scheefhals, H. D. MacGillavry, Membrane trafficking and positioning of mGluRs at presynaptic and postsynaptic sites of excitatory synapses. Neuropharmacology 200, 108799 (2021). 34592242 10.1016/j.neuropharm.2021.108799

[20] Q. Yan, M. J. Radeke, C. R. Matheson, J. Talvenheimo, A. A. Welcher, S. C. Feinstein, Immunocytochemical localization of TrkB in the central nervous system of the adult rat. J. Comp. Neurol. 378, 135–157 (1997). 9120052

[21] R. A. Wright, B. G. Johnson, C. Zhang, C. Salhoff, A. E. Kingston, D. O. Calligaro, J. A. Monn, D. D. Schoepp, G. J. Marek, CNS distribution of metabotropic glutamate 2 and 3 receptors: Transgenic mice and [^3^H]LY459477 autoradiography. Neuropharmacology 66, 89–98 (2013). 22313530 10.1016/j.neuropharm.2012.01.019

[22] L. E. Kilpatrick, S. J. Hill, Transactivation of G protein-coupled receptors (GPCRs) and receptor tyrosine kinases (RTKs): Recent insights using luminescence and fluorescence technologies. Curr. Opin. Endocr. Metab. Res. 16, 102–112 (2021). 33748531 10.1016/j.coemr.2020.10.003 PMC7960640

[23] Q. Wan, N. Okashah, A. Inoue, R. Nehmé, B. Carpenter, C. G. Tate, N. A. Lambert, Mini G protein probes for active G protein–coupled receptors (GPCRs) in live cells. J. Biol. Chem. 293, 7466–7473 (2018). 29523687 10.1074/jbc.RA118.001975 PMC5949987

[24] A. Castañé, N. Santana, F. Artigas, PCP-based mice models of schizophrenia: Differential behavioral, neurochemical and cellular effects of acute and subchronic treatments. Psychopharmacology 232, 4085–4097 (2015). 25943167 10.1007/s00213-015-3946-6

[25] R. Corbett, Animal models of negative symptoms M100907 antagonizes PCP-induced immobility in a forced swim test in mice. Neuropsychopharmacology 21 (Suppl. 2), S211–S218 (1999).

[26] K. Kawaura, J. Karasawa, H. Hikichi, Stimulation of the metabotropic glutamate (mGlu) 2 receptor attenuates the MK-801-induced increase in the immobility time in the forced swimming test in rats. Pharmacol. Rep. 68, 80–84 (2016). 26721357 10.1016/j.pharep.2015.05.027

[27] R. D. Paz, S. Tardito, M. Atzori, K. Y. Tseng, Glutamatergic dysfunction in schizophrenia: From basic neuroscience to clinical psychopharmacology. Eur. Neuropsychopharmacol. 18, 773–786 (2008). 18650071 10.1016/j.euroneuro.2008.06.005 PMC2831778

[28] Y. Tanabe, M. Masu, T. Ishii, R. Shigemoto, S. Nakanishi, A family of metabotropic glutamate receptors. Neuron 8, 169–179 (1992). 1309649 10.1016/0896-6273(92)90118-w

[29] S. Yin, M. J. Noetzel, K. A. Johnson, R. Zamorano, N. Jalan-Sakrikar, K. J. Gregory, P. J. Conn, C. M. Niswender, Selective actions of novel allosteric modulators reveal functional heteromers of metabotropic glutamate receptors in the CNS. J. Neurosci. 34, 79–94 (2014). 24381270 10.1523/JNEUROSCI.1129-13.2014 PMC3866496

[30] J. Lee, H. Munguba, V. A. Gutzeit, D. R. Singh, M. Kristt, J. S. Dittman, J. Levitz, Defining the Homo- and Heterodimerization Propensities of Metabotropic Glutamate Receptors. Cell Rep. 31, 107605 (2020). 32375054 10.1016/j.celrep.2020.107605 PMC7271767

[31] J. González-Maeso, R. L. Ang, T. Yuen, P. Chan, N. V. Weisstaub, J. F. López-Giménez, M. Zhou, Y. Okawa, L. F. Callado, G. Milligan, J. A. Gingrich, M. Filizola, J. J. Meana, S. C. Sealfon, Identification of a serotonin/glutamate receptor complex implicated in psychosis. Nature 452, 93–97 (2008). 18297054 10.1038/nature06612 PMC2743172

[32] S. A. Gaitonde, J. González-Maeso, Contribution of heteromerization to G protein-coupled receptor function. Curr. Opin. Pharmacol. 32, 23–31 (2017). 27835800 10.1016/j.coph.2016.10.006 PMC5395348

[33] M. Fribourg, J. L. Moreno, T. Holloway, D. Provasi, L. Baki, R. Mahajan, G. Park, S. K. Adney, C. Hatcher, J. M. Eltit, J. D. Ruta, L. Albizu, Z. Li, A. Umali, J. Shim, A. Fabiato, A. D. MacKerell, V. Brezina, S. C. Sealfon, M. Filizola, J. González-Maeso, D. E. Logothetis, Decoding the signaling of a GPCR heteromeric complex reveals a unifying mechanism of action of antipsychotic drugs. Cell 147, 1011–1023 (2011). 22118459 10.1016/j.cell.2011.09.055 PMC3255795

[34] K. S. Hideshima, A. Hojati, J. M. Saunders, D. M. On, M. de la Fuente Revenga, J. M. Shin, A. Sánchez-González, C. M. Dunn, A. B. Pais, A. C. Pais, M. F. Miles, J. T. Wolstenholme, J. González-Maeso, Role of mGlu2 in the 5-HT2A receptor-dependent antipsychotic activity of clozapine in mice. Psychopharmacology 235, 3149–3165 (2018). 30209534 10.1007/s00213-018-5015-4 PMC6408231

[35] A. Banerjee, R. S. Larsen, B. D. Philpot, O. Paulsen, Roles of presynaptic NMDA receptors in neurotransmission and plasticity. Trends Neurosci. 39, 26–39 (2016). 26726120 10.1016/j.tins.2015.11.001 PMC4716805

[36] J. Ster, J. M. Mateos, B. F. Grewe, G. Coiret, C. Corti, M. Corsi, F. Helmchen, U. Gerber, Enhancement of CA3 hippocampal network activity by activation of group II metabotropic glutamate receptors. Proc. Natl. Acad. Sci. U.S.A. 108, 9993–9997 (2011). 21628565 10.1073/pnas.1100548108 PMC3116403

[37] J. Jentsch, R. H. Roth, The Neuropsychopharmacology of Phencyclidine From NMDA Receptor Hypofunction to the Dopamine Hypothesis of Schizophrenia. Neuropsychopharmacology 20, 201–225 (1999). 10063482 10.1016/S0893-133X(98)00060-8

[38] A. Pillai, Brain-derived neurotropic factor/TrkB signaling in the pathogenesis and novel pharmacotherapy of schizophrenia. Neurosignals 16, 183–193 (2008). 18253057 10.1159/000111562

[39] Y.-J. Yang, Y.-K. Li, W. Wang, J.-G. Wan, B. Yu, M.-Z. Wang, B. Hu, Small-molecule TrkB agonist 7,8-dihydroxyflavone reverses cognitive and synaptic plasticity deficits in a rat model of schizophrenia. Pharmacol. Biochem. Behav. 122, 30–36 (2014). 24662915 10.1016/j.pbb.2014.03.013

[40] S. L. O’Brien, E. K. M. Johnstone, D. Devost, J. Conroy, M. E. Reichelt, B. W. Purdue, M. A. Ayoub, T. Kawai, A. Inoue, S. Eguchi, T. E. Hébert, K. D. G. Pfleger, W. G. Thomas, BRET-based assay to monitor EGFR transactivation by the AT1R reveals Gq/11 protein-independent activation and AT1R-EGFR complexes. Biochem. Pharmacol. 158, 232–242 (2018). 30347205 10.1016/j.bcp.2018.10.017 PMC8491139

[41] N. J. Smith, H.-W. Chan, H. Qian, A. M. Bourne, K. M. Hannan, F. J. Warner, R. H. Ritchie, R. B. Pearson, R. D. Hannan, W. G. Thomas, Determination of the exact molecular requirements for type 1 angiotensin receptor epidermal growth factor receptor transactivation and cardiomyocyte hypertrophy. Hypertension 57, 973–980 (2011). 21383310 10.1161/HYPERTENSIONAHA.110.166710

[42] F. S. Lee, M. V. Chao, Activation of Trk neurotrophin receptors in the absence of neurotrophins. Proc. Natl. Acad. Sci. U.S.A. 98, 3555–3560 (2001). 11248116 10.1073/pnas.061020198 PMC30691

[43] F. S. Lee, R. Rajagopal, A. H. Kim, P. C. Chang, M. V. Chao, Activation of Trk neurotrophin receptor signaling by pituitary adenylate cyclase-activating polypeptides. J. Biol. Chem. 277, 9096–9102 (2002). 11784714 10.1074/jbc.M107421200

[44] Y. Hu, Q. Sun, W. Zhang, Y. Huo, C. Xu, J. Liu, Specific activation of mGlu2 induced IGF-1R transactivation in vitro through FAK phosphorylation. Acta Pharmacol. Sin. 40, 460–467 (2019). 29946167 10.1038/s41401-018-0033-7 PMC6461959

[45] N. Delcourt, J. Bockaert, P. Marin, GPCR-jacking: From a new route in RTK signalling to a new concept in GPCR activation. Trends Pharmacol. Sci. 28, 602–607 (2007). 18001849 10.1016/j.tips.2007.09.007

[46] C. S. Wang, E. T. Kavalali, L. M. Monteggia, BDNF signaling in context: From synaptic regulation to psychiatric disorders. Cell 185, 62–76 (2022). 34963057 10.1016/j.cell.2021.12.003 PMC8741740

[47] P. C. Casarotto, M. Girych, S. M. Fred, V. Kovaleva, R. Moliner, G. Enkavi, C. Biojone, C. Cannarozzo, M. P. Sahu, K. Kaurinkoski, C. A. Brunello, A. Steinzeig, F. Winkel, S. Patil, S. Vestring, T. Serchov, C. R. A. F. Diniz, L. Laukkanen, I. Cardon, H. Antila, T. Rog, T. P. Piepponen, C. R. Bramham, C. Normann, S. E. Lauri, M. Saarma, I. Vattulainen, E. Castrén, Antidepressant drugs act by directly binding to TRKB neurotrophin receptors. Cell 184, 1299–1313.e19 (2021). 33606976 10.1016/j.cell.2021.01.034 PMC7938888

[48] C. Dong, J. Zhang, W. Yao, Q. Ren, M. Ma, C. Yang, S. Chaki, K. Hashimoto, Rapid and sustained antidepressant action of the mGlu2/3 receptor antagonist MGS0039 in the social defeat stress model: Comparison with ketamine. Int. J. Neuropsychopharmacol. 20, 228–236 (2017). 27765808 10.1093/ijnp/pyw089 PMC5408970

[49] A. Rafało-Ulińska, P. Brański, A. Pałucha-Poniewiera, Combined administration of (*R*)-ketamine and the mGlu2/3 receptor antagonist LY341495 induces rapid and sustained effects in the CUMS model of depression via a TrkB/BDNF-dependent mechanism. Pharmaceuticals (Basel) 15, 125 (2022). 35215237 10.3390/ph15020125 PMC8879988

[50] A. S. Tsybko, T. V. Ilchibaeva, E. A. Filimonova, D. V. Eremin, N. K. Popova, V. S. Naumenko, The chronic treatment with 5-HT2A receptor agonists affects the behavior and the BDNF system in mice. Neurochem. Res. 45, 3059–3075 (2020). 33095437 10.1007/s11064-020-03153-5

[51] A. Pillai, A. V. Terry, S. P. Mahadik, Differential effects of long-term treatment with typical and atypical antipsychotics on NGF and BDNF levels in rat striatum and hippocampus. Schizophr. Res. 82, 95–106 (2006). 16442781 10.1016/j.schres.2005.11.021

[52] R. J. Lefkowitz, K. L. Pierce, L. M. Luttrell, Dancing with different partners: Protein kinase A phosphorylation of seven membrane-spanning receptors regulates their G protein-coupling specificity. Mol. Pharmacol. 62, 971–974 (2002). 12391258 10.1124/mol.62.5.971

[53] C. N. Pujol, V. Dupuy, M. Séveno, L. Runtz, J. Bockaert, P. Marin, S. Chaumont-Dubel, Dynamic interactions of the 5-HT_6_ receptor with protein partners control dendritic tree morphogenesis. Sci. Signal. 13, eaax9520 (2020). 32047117 10.1126/scisignal.aax9520

[54] S. Murat, M. Bigot, J. Chapron, G. M. König, E. Kostenis, G. Battaglia, F. Nicoletti, E. Bourinet, J. Bockaert, P. Marin, F. Vandermoere, 5-HT2A receptor-dependent phosphorylation of mGlu2 receptor at Serine 843 promotes mGlu2 receptor-operated Gi/o signaling. Mol. Psychiatry 24, 1610–1626 (2019). 29858599 10.1038/s41380-018-0069-6

[55] D. O. Borroto-Escuela, M. Flajolet, L. F. Agnati, P. Greengard, K. Fuxe, Bioluminescence resonance energy transfer methods to study G protein-coupled receptor–receptor tyrosine kinase heteroreceptor complexes. Methods Cell Biol. 117, 141–164 (2013). 24143976 10.1016/B978-0-12-408143-7.00008-6 PMC3921556

[56] F. Jeanneteau, M. J. Garabedian, M. V. Chao, Activation of Trk neurotrophin receptors by glucocorticoids provides a neuroprotective effect. Proc. Natl. Acad. Sci. U.S.A. 105, 4862–4867 (2008). 18347336 10.1073/pnas.0709102105 PMC2290769

[57] I. Sebastianutto, E. Goyet, L. Andreoli, J. Font-Ingles, D. Moreno-Delgado, N. Bouquier, C. Jahannault-Talignani, E. Moutin, L. Di Menna, N. Maslava, J.-P. Pin, L. Fagni, F. Nicoletti, F. Ango, M. A. Cenci, J. Perroy, D1-mGlu5 heteromers mediate noncanonical dopamine signaling in Parkinson’s disease. J. Clin. Investig. 130, 1168–1184 (2020). 32039920 10.1172/JCI126361 PMC7269571

[58] M. Leger, A. Quiedeville, V. Bouet, B. Haelewyn, M. Boulouard, P. Schumann-Bard, T. Freret, Object recognition test in mice. Nat. Protoc. 8, 2531–2537 (2013). 24263092 10.1038/nprot.2013.155

