# Supplementary material for: TrkB receptor interacts with mGlu2 receptor and mediates antipsychotic-like effects of mGlu2 receptor activation in the mouse
Source: Sci Adv. 2024 Jan 26;10(4):eadg1679. doi: 10.1126/sciadv.adg1679 (PMC10816717; doi:10.1126/sciadv.adg1679)
Supplement: Supplementary file 1 — Figs. S1 to S9 Legends for tables S1 and S2 [file sciadv.adg1679_sm.pdf]

Science Advances
AAAS

Supplementary Materials for

TrkB receptor interacts with mGlu$_2$ receptor and mediates antipsychotic-like effects of mGlu$_2$ receptor activation in the mouse

Clémentine Eva Philibert *et al.*

Corresponding author: Clémentine Eva Philibert, philibert.clementine@gmail.com;
Philippe Marin, philippe.marin@igf.cnrs.fr; Franck Vandermoere, franck.vandermoere@cnrs.fr

Sci. Adv. **10**, eadg1679 (2024)
DOI: 10.1126/sciadv.adg1679

The PDF file includes:

Figs. S1 to S9
Legends for tables S1 and S2

Other Supplementary Material for this manuscript includes the following:

Tables S1 and S2

Fig. S1.

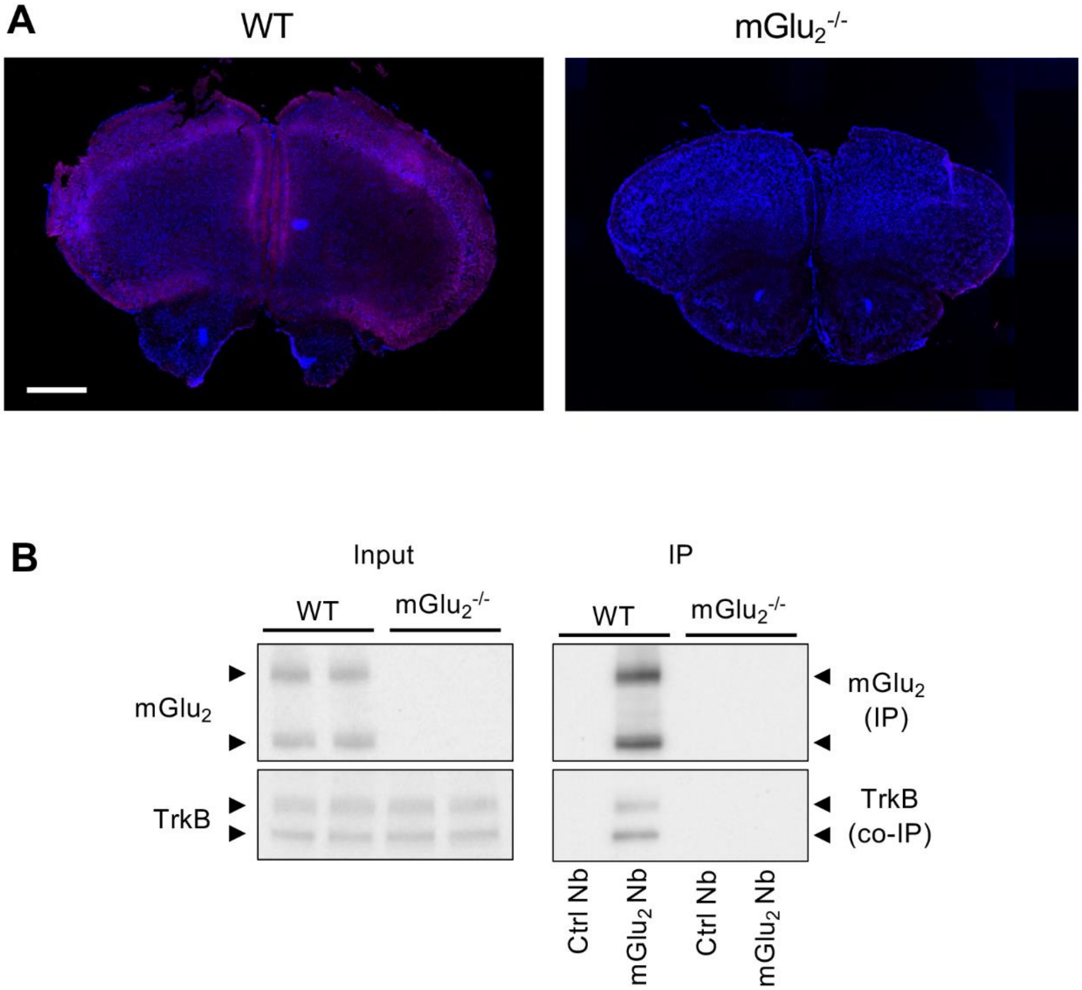

Specificity of the mGlu$_2$ nanobody used in the interactomics screen. A, Immunofluorescence labeling of mGlu$_2$ receptors with the mGlu$_2$ nanobody in coronal sections from wild-type and mGlu$_2^{-/-}$ mouse prefrontal cortex. The nanobody signal is shown in magenta and cell nuclei are counterstained with DAPI (blue). Scale bar: 1 mm. **B,** Prefrontal cortex protein extracts from wild-type and mGlu$_2^{-/-}$ mice were immunoprecipitated with either the mGlu$_2$ nanobody or a control non-immune nanobody. Immunoprecipitated mGlu$_2$ and TrkB were assessed by immunoblotting. The input represents 5% of the protein amount used for immunoprecipitations. Immunoblots representative of two independent experiments are illustrated.

Fig. S2.

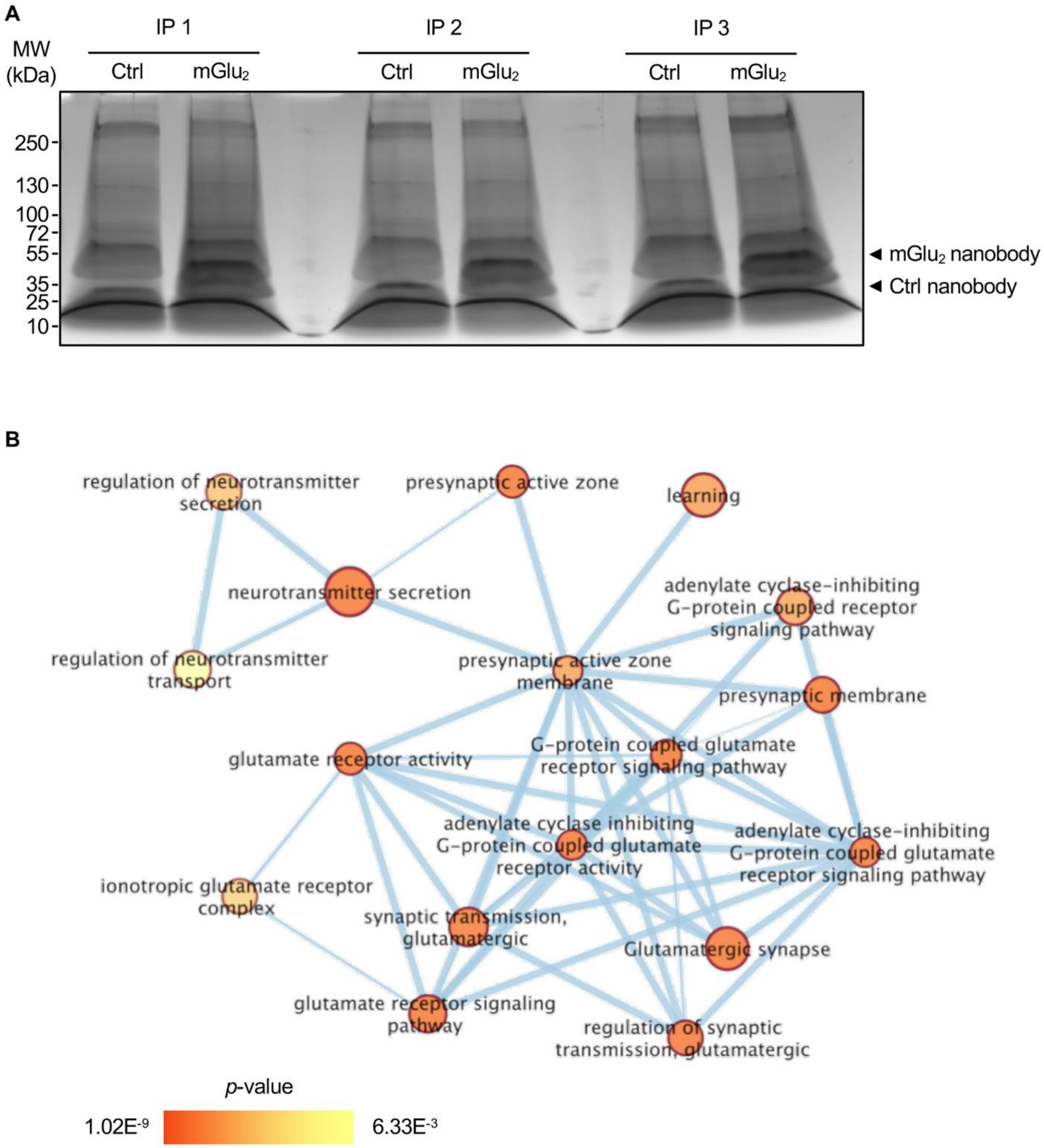

Enrichment of gene ontology (GO) annotations in mGlu$_2$ interactome. A, Colloidal-blue stained gel of nanobody-based immunoprecipitation of mGlu$_2$ from the mouse prefrontal cortex. Immunoprecipitation with the control nanobody (Ctrl) or the anti-mGlu$_2$ nanobody (mGlu$_2$) in three different mice. Each mouse was used as its own control**. B,** The most enriched molecular function, biological process and cellular component GO annotations are illustrated using the

Cytoscape software. The connector thickness corresponds to the number of genes common to the connected annotations. Size and color of nodes correspond to the statistical significance of GO annotation enrichment in the $mGlu_2$ interactome ($-\log_{10}$ of *p*-value, one-sided Student's t test).

Fig. S3.

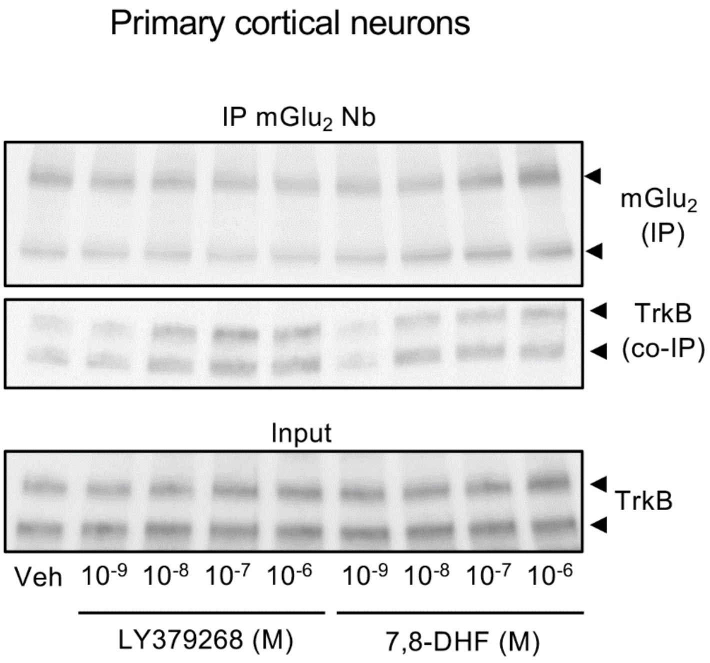

Effect of agonist stimulation of $mGlu_2$ and TrkB on $mGlu_2$ association with TrkB. Primary cultured cortical neurons were exposed to either vehicle or the indicated concentrations of LY379268 or 7,8-DHF for 15 min. Neuronal protein extracts were immunoprecipitated with the $mGlu_2$ nanobody. Immunoprecipitated $mGlu_2$ and TrkB were assessed by immunoblotting. Equivalent TrkB expression in neuronal protein extracts (Input) in the different experimental conditions was assessed by TrkB immunoblotting. The input represents 5% of the protein amount used for immunoprecipitations. Immunoblots representative of two independent experiments are illustrated.

Fig. S4.

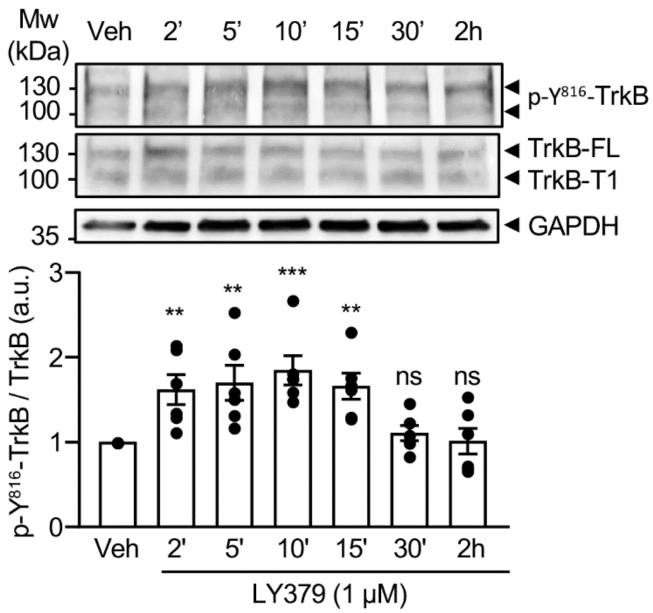

TrkB activation by LY379268 in primary cultures of cortical neurons. Representative Western blots showing TrkB phosphorylation on Tyr^{816} in lysates from cortical neurons upon exposure to LY379268 (1 μM) for increasing times. The histogram represents the means ± SEM of anti-p-Y^{816}-TrkB immunoreactive signal relative to anti-TrkB immunoreactive signal (in arbitrary units, a.u., n=4). One-way ANOVA (post-hoc: Dunnett's test): $F(6, 35) = 1.748$.

Fig. S5.

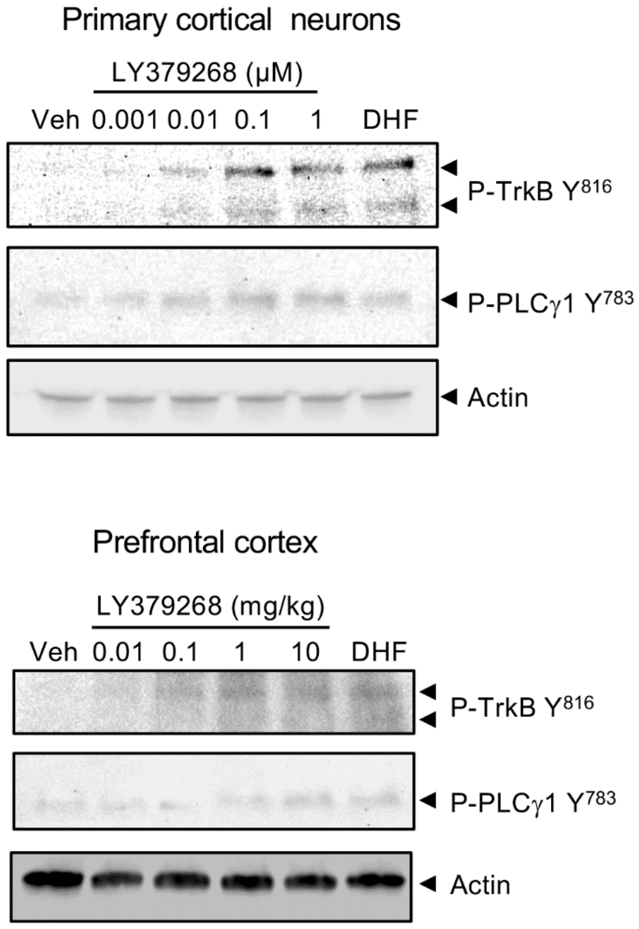

Effects of LY379268 on phosphorylation of TrkB and its substrate PLCγ1. Top panels Western blots assessing p-Y^{816}-TrkB and p-PLCγ1-Y^{783} in primary cultures of cortical neurons exposed to 0.001 to 1 µM LY379268 or 1 µM 7,8-DHF for 15 min. **Bottom panels** Western blots assessing p-Y^{816}-TrkB and p-PLCγ1-Y^{783} in prefrontal cortex protein extracts from wild type mice injected with 0.01 to 10 mg/kg LY379268 or 10 mg/kg DHF and sacrificed 30 min after the injection of drugs. Actin immunoblotting was performed to assess equal protein loading in each lane. Immunoblots representative of two independent experiments are illustrated.

Fig. S6.

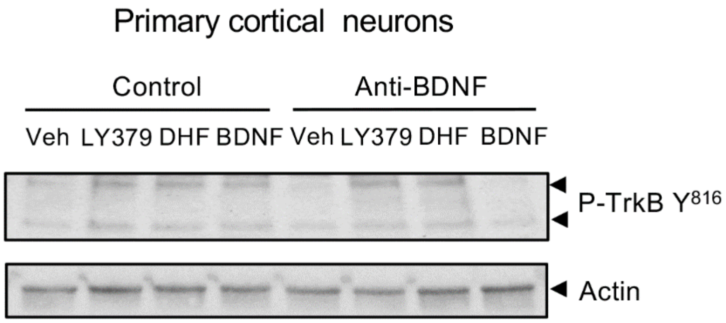

A neutralizing BDNF antibody prevents TrkB phosphorylation induced by BDNF but not those induced by LY379268 of 7,8-DHF in cortical neurons. Primary cultured cortical neurons were preincubated for 5 min with vehicle (5 µg/mL BSA, Control) or with a neutralizing BDNF antibody (anti-BDNF, 5 µg/mL) and then exposed to either Vehicle or 1 µM LY379268 or 1 µM 7,8-DHF or 10 nM BDNF for 15 min. Immunoblots for p-Y^{816}-TrkB and actin (to assess equal protein loading in each lane) representative of two independent experiments are illustrated.

Fig. S7.

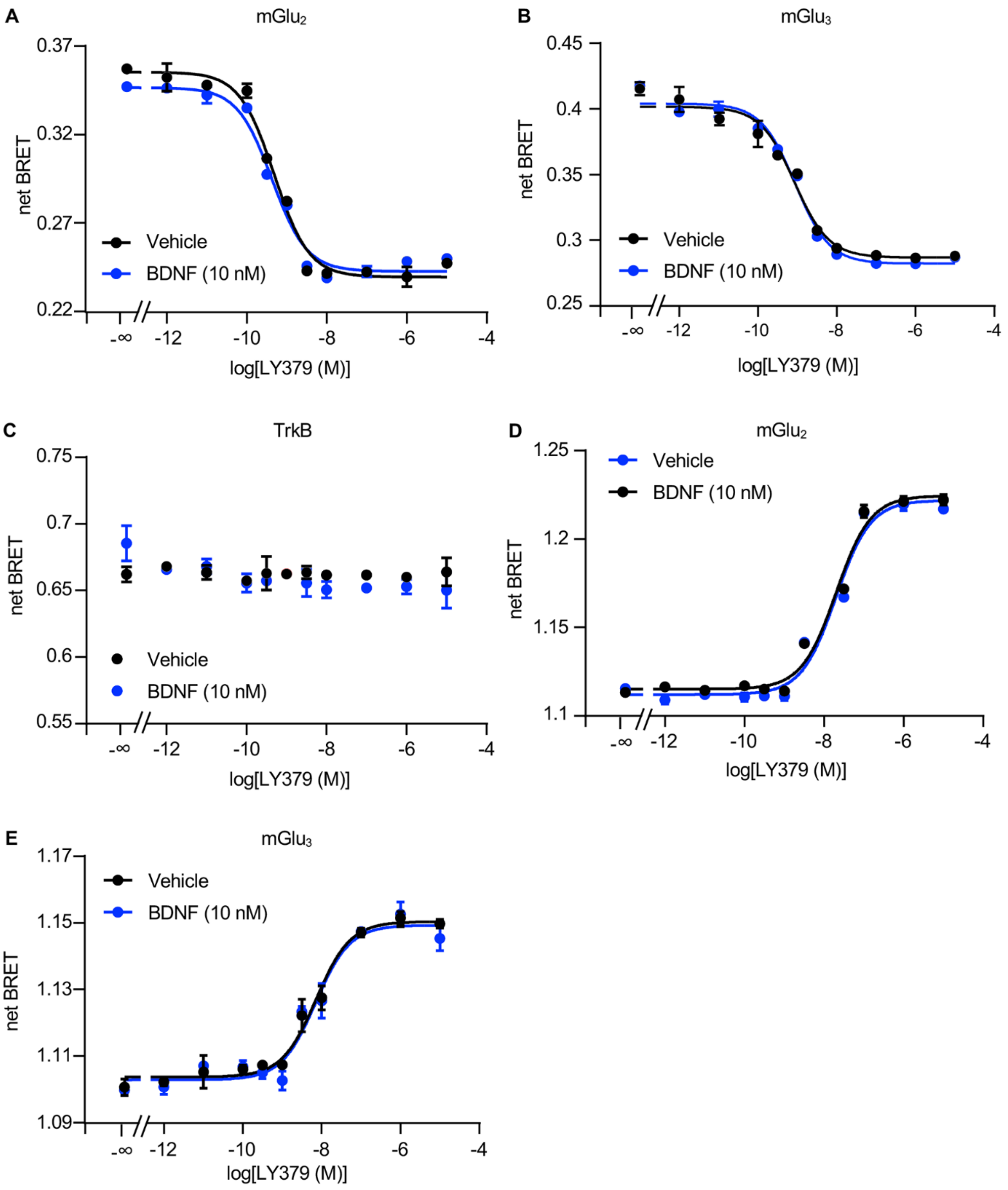

Negative controls for BRET experiments of Fig. 5. A,B,C, $G\alpha_o$ and $G\beta\gamma$ dissociation induced by increasing concentrations of LY379268 in presence or absence of BDNF (10 nM, blue) in HEK-293 cells co-expressing $G\alpha_{oA}$-Rluc and $G\gamma$-Venus, with either human Flag-mGlu$_2$ (**A**) or Flag-mGlu$_3$ (**B**) or TrkB (**C**). The curves show the means ± SEM of net BRET ratios in three technical replicates of one experiment representative of three independent biological replicates performed

on different sets of cultured cells. Two-way ANOVA (post-hoc: Sidak's multiple comparison test): $mGlu_2$ $F(10,22)=553.5$, $mGlu_3$ $F(10,43)=350.0$, TrkB $F(10,22)=1.411$. **D,E,** Representative BRET assay assessing miniGα_i recruitment by $mGlu_2$ or $mGlu_3$ induced by increasing concentrations of LY379268 in presence or absence of BDNF (10 nM) in HEK-293 cells expressing miniGα_i-Rluc protein and either human $mGlu_2$-Venus (**D**) or $mGlu_3$-Venus (**E**), respectively. Two-way ANOVA (post-hoc: Sidak's multiple comparison test): $mGlu_2$ $F(10,43)=1108.0$, $mGlu_3$ $F(10,22)=116.1$.

Fig. S8.

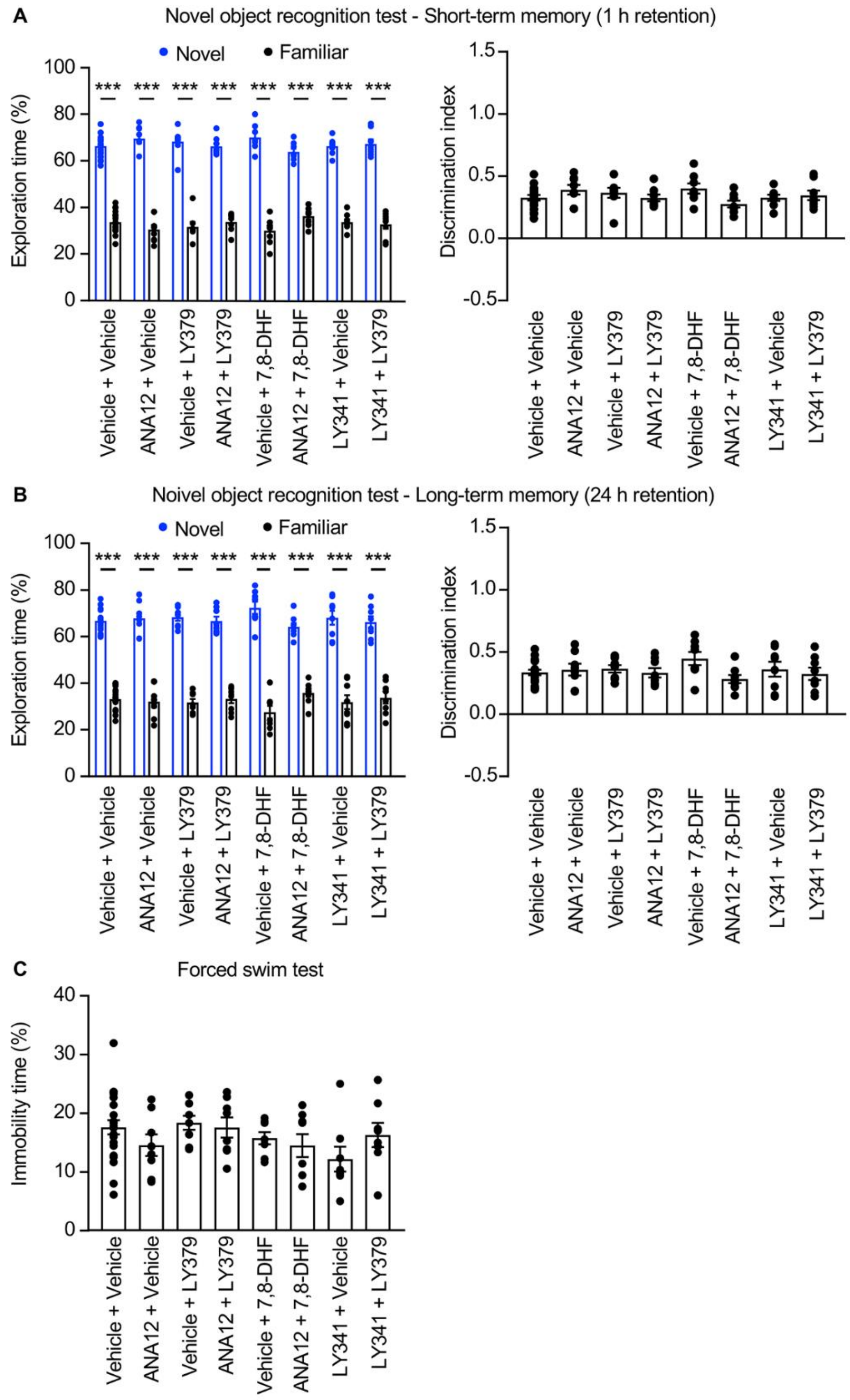

Effect of mGlu2 and TrkB activation on recognition memory and resignation in control mice. A,B, Mice were pre-treated with either vehicle or ANA12 (2.5 mg/kg) or LY341495 (3 mg/kg, LY341) and then injected with either vehicle or LY379268 (10 mg/kg, LY379) or 7,8-DHF (5 mg/kg). The left histograms represent the means ± SEM of the percentage of exploration time of

novel *vs.* familiar object. The right histograms represent the means ± SEM of the discrimination index measured in each group including more than eight mice. Kruskal-Wallis test (multiple comparisons compared to PCP Vehicle, post-hoc: Dunn's test): *** $p < 0.001$. **C,** Mice were treated as in (**A,B**) and subjected to the forced swim test. The histogram represents the percentage of immobility during the test. Kruskal-Wallis test (multiple comparisons compared to PCP Vehicle, post-hoc: Dunn's test): $p > 0.05$

Fig. S9.

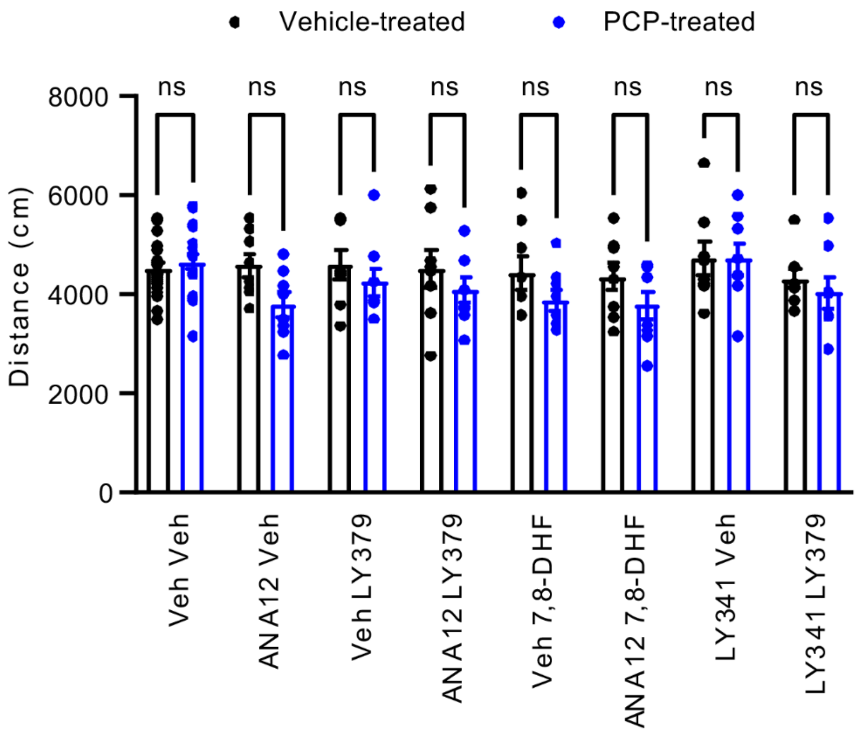

Effect of mGlu2 and TrkB activation on locomotion. Mice were pre-treated with either vehicle or ANA12 (2.5 mg/kg) or LY341495 (3 mg/kg, LY341) and then injected with either vehicle or LY379268 (10 mg/kg, LY379) or 7,8-DHF (5 mg/kg). The histogram represents the means ± SEM of the distance traveled by the mice in each group including more than eight mice. Two-way ANOVA (post hoc: Šídák's multiple comparisons). $p \geq 0.05$

Table S1.

List of proteins that specifically co-immunoprecipitate with $mGlu_2$ in mice prefrontal cortex. The table provides detailed information on proteins statistically enriched in $mGlu_2$ immunoprecipitates: protein name, UniprotKB accession number, gene name, fold of enrichment (ratio of MS protein intensities in $mGlu_2$ *vs.* control immunoprecipitates), the $-\log_{10}$(*p*-value) of t test for difference in protein MS intensity in $mGlu_2$ vs. Ctrl immunoprecipitates and the raw protein MS intensity in each replicate. Proteins are ranked based on their statistical relevance ($-\log_{10}$(*p*-value)). The bait $mGlu_2$ (GRM2) is shown in pink, proteins already known to interact with $mGlu_2$ in blue and TrkB (NTRK2) in green.

Table S2.

Gene ontology (GO) annotations enriched in $mGlu_2$ interactome. The list of genes corresponding to proteins of $mGlu_2$ interactome was processed in g:Profiler software in order to look for GO annotation enrichments. Molecular function (MF), biological process (BP), cellular component (CC) GO annotations significantly enriched in $mGlu_2$ interactome are listed. GO term names, ID, *p*-value of enrichment, GO term size (numbers of proteins that match the annotation term in the *Mus musculus* database), query size, intersection size (number of hits in the input list), effective domain site (GO reference number) and the list of gene names that matched the GO term (intersections) are indicated.
